# Revealing the Local Structure and Dynamics of the Solid Li Ion Conductor Li_3_P_5_O_14_

**DOI:** 10.1021/acs.chemmater.4c00727

**Published:** 2024-07-29

**Authors:** Benjamin B. Duff, Lucia Corti, Bethan Turner, Guopeng Han, Luke M. Daniels, Matthew J. Rosseinsky, Frédéric Blanc

**Affiliations:** † Department of Chemistry, University of Liverpool, L69 7ZD Liverpool, U.K.; ‡ Stephenson Institute for Renewable Energy, University of Liverpool, L69 7ZF Liverpool, U.K.; § Leverhulme Research Centre for Functional Materials Design, Materials Innovation Factory, University of Liverpool, L7 3NY Liverpool, United Kingdom

## Abstract

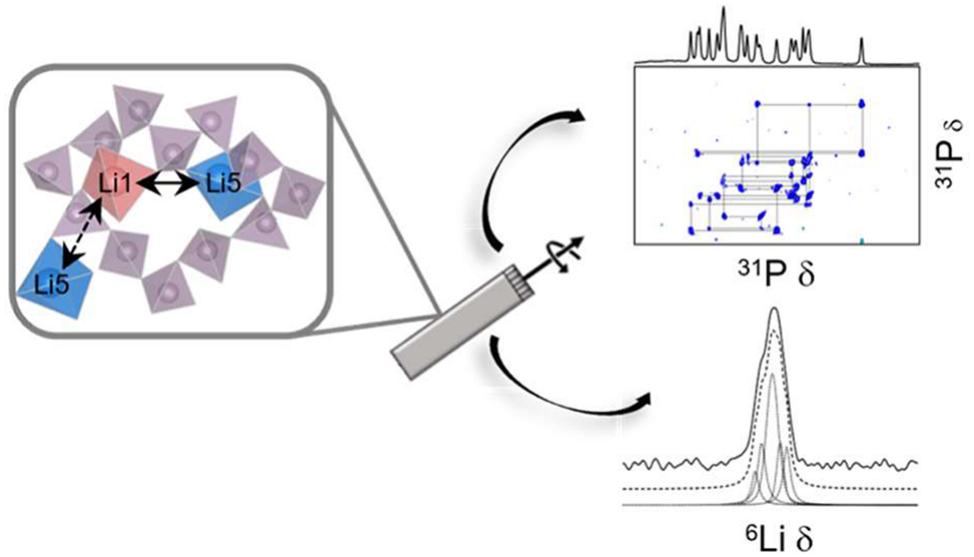

The development of fast Li ion-conducting materials for use as solid electrolytes that provide sufficient electrochemical stability against electrode materials is paramount for the future of all-solid-state batteries. Advances on these fast ionic materials are dependent on building structure-ionic mobility-function relationships. Here, we exploit a series of multinuclear and multidimensional nuclear magnetic resonance (NMR) approaches, including ^6^Li and ^31^P magic angle spinning (MAS), in conjunction with density functional theory (DFT) to provide a detailed understanding of the local structure of the ultraphosphate Li_3_P_5_O_14_, a promising candidate for an oxide-based Li ion conductor that has been shown to be a highly conductive, energetically favorable, and electrochemically stable potential solid electrolyte. We have reported a comprehensive assignment of the ultraphosphate layer and layered Li_6_O_16_^26–^ chains through ^31^P and ^6^Li MAS NMR, respectively, in conjunction with DFT. The chemical shift anisotropy of the eight resonances with the lowest ^31^P chemical shift is significantly lower than that of the 12 remaining resonances, suggesting the phosphate bonding nature of these P sites being one that bridges to three other phosphate groups. We employed a number of complementary ^6,7^Li NMR techniques, including MAS variable-temperature line narrowing spectra, spin-alignment echo (SAE) NMR, and relaxometry, to quantify the lithium ion dynamics in Li_3_P_5_O_14_. Detailed analysis of the diffusion-induced spin-lattice relaxation data allowed for experimental verification of the three-dimensional Li diffusion previously proposed computationally. The ^6^Li NMR relaxation rates suggest sites Li1 and Li5 (the only five-coordinate Li site) are the most mobile and are adjacent to one another, both in the *a-b* plane (intralayer) and on the *c*-axis (interlayer). As shown in the ^6^Li-^6^Li exchange spectroscopy NMR spectra, sites Li1 and Li5 likely exchange with one another both between adjacent layered Li_6_O_16_^26–^ chains and through the center of the P_12_O_36_^12–^ rings forming the three-dimensional pathway. The understanding of the Li ion mobility pathways in high-performing solid electrolytes outlines a route for further development of such materials to improve their performance.

## Introduction

Significant progress has been made in the field of next-generation lithium ion batteries with a significant emphasis on the implantation of solid-state electrolytes (SSEs) for the generation of all-solid-state batteries (ASSBs). The target of a room-temperature ionic conductivity of 10^–3^ S cm^–1^ has now been met in a range of sulfide-based SSEs.^1−5^ However, sulfide-based materials generally are not stable under atmospheric conditions and react with water in the atmosphere to generate H_2_S; furthermore, sulfides often do not form suitable interfaces with electrodes.^6−8^ In contrast, oxide-based lithium ion conductors tend to have lower total ionic conductivities but improved stabilities. Phosphate-based lithium ion conductors provide an alternative avenue for meeting the conductivity target, while maintaining the required stability for an ASSB. Additionally, phosphate-based ionic conductors are considered among the most promising candidates for cathode coatings,^9−11^ providing a buffer between a highly conductive SSE and the electrode, allowing for increased stability and performance.

**Figure 1:**
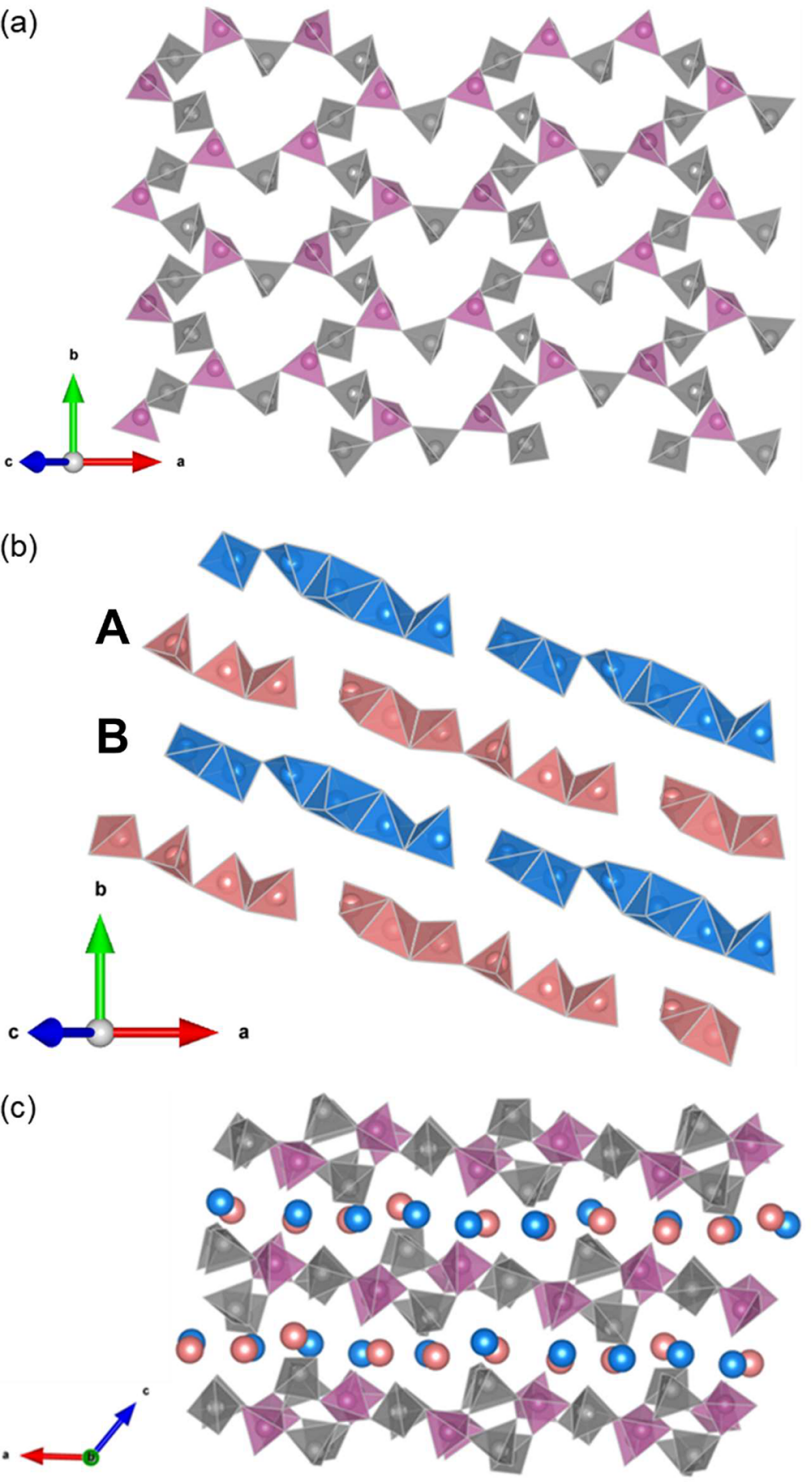
Crystal structure of Li_3_P_5_O_14_ and polyhedral arrangement of Li and P. (a) Arrangement of the infinite P_20_O_56_^12–^ ultraphosphate layers in Li_3_P_5_O_14_ in which gray and purple tetrahedra correspond to PO_4_^3–^ units that branch to two and three other PO_4_^3–^ tetrahedra, respectively. (b) Arrangement of lithium in Li_3_P_5_O_14_, the two types of Li_6_O_16_^26–^ chains, type A (red) and type B (blue), with different connection modes along with two distinct vacant tetrahedral sites at the terminating ends and the corresponding stacking of the Li_6_O_16_^26–^ chains viewed along the [110] direction. (c) Projection of the atomic arrangement in Li_3_P_5_O_14_ along the *b*-axis, showing P_20_O_56_^12–^ ultraphosphate layers alternately stacked with Li polyhedral layers. The gray and purple tetrahedra represent internal and branching PO_4_^3–^ tetrahedra, respectively, while Li atoms are represented by blue and red spheres.

Orthophosphate, polyphosphate, cyclophosphate, and ultraphosphate structures are a result of PO_4_^3–^ tetrahedra adopting isolated, linear, cyclic, and branched anionic substructures, respectively.^12,13^ In the first three of these structural families, PO_4_^3–^ tetrahedra share zero (isolated tetrahedra), one (terminal tetrahedra), or two (internal tetrahedra) of their oxygens with neighboring tetrahedra, resulting in unbranched 0-, 2-, or mixed 1,2-connected anions, so only unbranched zero-dimensional (0D) or one-dimensional (1D) anions are available, e.g., 0D unbranched 2-connected single P_6_O_18_^6–^ rings in the cyclophosphate Al_2_P_6_O_18_^14^ and 1D 2-connected PO_3_^–^ chains in the polyphosphate LiPO_3_,^15^ as shown in Figure S1. In contrast, upon combination of internal tetrahedra with branching PO_4_^3–^ tetrahedra that share three of their oxygens with other tetrahedra, ultraphosphates show branched anions that create a two-dimensional (2D) structure of linked PO_4_^3–^ tetrahedra (Figure 1a). This results in 2,3-connected nets that lie between the 2-connected cyclophosphates and the purely 3-connected phosphoric anhydride P_2_O_5_, producing richer structural chemistry due to topologically nonlinear linking. The arising anion geometries are more diverse than in other types of phosphates; e.g., ultraphosphates could adopt 0D (finite P_8_O_23_^6–^ groups in Na_3_FeP_8_O_23_),^16^ 1D (infinite P_5_O_14_^3–^ ribbons in orthorhombic HoP_5_O_14_),^17^ 2D (P_4_O_11_^2–^ layers in CaP_4_O_11_),^18^ or even three-dimensional (3D) [infinite P_6_O_17_^4–^ frameworks in (UO_2_)_2_P_6_O_17_]^19^ anionic geometries.

In this context, we recently discovered Li_3_P_5_O_14_, which possesses layers of the 12-membered ring ultraphosphate motif stacked alternately with lithium polyhedral layers and is the most crystallographically complex lithium phosphate known. This phase shows a promising room-temperature lithium ion conductivity of 8.5(5) × 10^–7^ S cm^–1^, the highest of any reported ternary Li-P-O phases, along with the lowest activation energy of 0.43(7) eV.^20^ Moreover, this newly reported ultraphosphate phase is predicted to have a high thermodynamic stability against oxidation, with a predicted stability up to 4.8 V. The ultraphosphate layers in Li_3_P_5_O_14_ produce a unique topology for the Li sublattices: two types of finite Li polyhedral Li_6_O_16_^26–^ chains, isolated from each other, with comparatively short Li-Li distances (2.581-3.235 Å in the Li_6_O_16_^26–^ chains) terminated with two distinct vacant tetrahedral sites (Figure 1b). The type A Li_6_O_16_^26–^ chain consists of six crystallographically distinct corner- and edge-shared distorted tetrahedra. The type B Li_6_O_16_^26–^ chain consists of five distorted tetrahedra and a Li5 distorted square pyramid connected by corner and edge sharing. These two types of Li_6_O_16_^26–^ chains are alternately arranged parallel to the *a-b* plane, forming Li polyhedral layers. These Li polyhedral layers are further alternately stacked with infinite ultraphosphate layers along the *c*-axis to form a 3D framework. Similar to the Li-occupied sites, the two vacant tetrahedra are coordinated by four PO_4_^3–^ tetrahedra by corner sharing (two internal PO_4_^3–^ tetrahedra and two branching PO_4_^3–^ tetrahedra) and two LiO_4_^7–^ tetrahedra by edge sharing.

Li_3_P_5_O_14_ is thus a layered structure built from infinite ultraphosphate P_20_O_56_^12–^ layers with 12-membered corrugated P_12_O_36_^12–^ rings constructed from corner-sharing PO_4_^3–^ tetrahedra (Figure 1a) alternately stacked with Li polyhedral layers along the *c*-axis (Figure 1c). The charge-compensating Li cations are located between P_20_O_56_^12–^ layers, coordinating to four or five oxide ions in these adjacent layers. The P_20_O_56_^12–^ layers provide pathways for the transport of ions between adjacent Li polyhedral layers. There are four crystallographically distinct P_12_O_36_^12–^ rings, which are similar in size and shape, and each is connected to six adjacent rings through branching PO_4_^3–^ tetrahedra to form an infinite ultraphosphate layer.

Because of the sensitivity of the interactions that affect the nuclear spin to the local environment, nuclear magnetic resonance (NMR) spectroscopy is an extremely powerful approach for understanding the short-range structure^21,22^ and accessing both ion dynamics and diffusion processes in potential SSE candidates.^23−25^ Magic angle spinning (MAS) NMR provides an isotope specific viewpoint of the local structure of materials that coupled with multidimensional NMR gives a comprehensive structural characterization of the short-range structures of a variety of species and has been successfully implemented in material science,^26,27^ chemistry,^28−30^ and biology.^31−34^ More specifically, NMR offers a nondestructive method for the direct observation of Li^+^ mobility by exploiting the two NMR active isotopes of Li (^6^Li, 7.59% natural abundance, spin *I* = 1; and ^7^Li, 92.41%, *I* = ^3^/_2_). While ^6^Li NMR spectra are often highly resolved, the sensitivity of this nuclear spin is challenged by both its low natural abundance and lower gyromagnetic ratio. In contrast, ^7^Li is very receptive but often suffers from poor resolution due to strong homonuclear dipolar broadening. An additional benefit of NMR in the investigation of ion dynamics is the range of motional processes that can be probed, from very fast motional processes on the order of 10^–12^ s^–1^ probed by measuring spin-lattice relaxation (SLR) time constants to much slower motion on the time scale of 10^–3^ s^–1^ from line shape analysis and 1 s^–1^ in exchange spectroscopy (EXSY) and spin-alignment echo (SAE) NMR. For example, ^7^Li NMR SLR rate constants in the laboratory (*T*_1_^–1^) and rotating (*T*_1ρ_^–1^) frames of reference give quantitative information about the Li ion mobility in SSEs along with the dimensionality of Li diffusion through the frequency dependence of the rate constants.^35,36^

In this work, we provide a comprehensive structural characterization of the local Li and P sites in the Li_6_O_16_^26–^ chains and P_20_O_56_^12–^ ultraphosphate layers in Li_3_P_5_O_14_ through a combined MAS and computational approach. Moreover, we experimentally capture the Li ion mobility both qualitatively and quantitatively to identify the 3D ion mobility pathway through the frequency dependence of the SLR data and present a potential rationale for the high ion mobility in this ultraphosphate SSE.

## Experimental Section

### Synthesis of Materials

Li_2_O (97%) and P_2_O_5_ (≥98.0%), purchased from Sigma-Aldrich, were dried overnight under a vacuum (10^–4^ mbar) at room temperature before being transferred into an Ar-filled glovebox. Li_3_P_5_O_14_ was synthesized according to the previously reported solid-state synthesis procedure from these Li_2_O and P_2_O_5_ reagents.^20^ All samples were handled in an Ar-filled glovebox (<0.1 ppm O_2_ and <0.1 ppm H_2_O).

### Solid-State MAS NMR Experiments

Room-temperature ^31^P and ^6^Li MAS NMR experiments were performed on a 9.4 T Bruker Avance III HD spectrometer using a 4 mm HXY MAS probe (in double-resonance mode) at a MAS frequency ω_r_/2π of 10 kHz with the X channel tuned to ^31^P and ^6^Li at ω_0_/2π (^31^P and ^6^Li) = 162 and 59 MHz, respectively. Room-temperature ^6^Li MAS experiments were one-pulse experiments, while ^31^P MAS experiments were one-pulse and Hahn echo sequences. The π/2 pulse durations of 3 and 3.8 μs at radiofrequency (rf) field amplitudes ω_1_/2π of 83 and 65 kHz were used for ^6^Li and ^31^P, respectively. The spinning side bands in the ^31^P MAS NMR spectra were fitted with solid line shape analysis tool “Sola” in Topspin, to extract chemical shift anisotropy (CSA) values that follow the Haeberlen convention (see below). Variable-temperature ^6^Li MAS experiments were performed on a 20 T Bruker Avance NEO spectrometer using a 4 mm HX high-temperature MAS probe at a MAS frequency ω_r_/2π of 10 kHz with the X channel tuned to ^6^Li at ω_0_/2π = 125 MHz. Spectra were recorded with π/2 pulse durations of 5 μs at an rf field amplitude ω_1_/2π of 50 kHz. All MAS experiments were performed with quantitative recycle delays of >5 times the ^6^Li and ^31^P longitudinal relaxation time, *T*_1_, measured via the saturation recovery pulse sequence (π/2–*d*)_x100_–τ–π/2–acq with *d* a short delay (1 ms) and increasing recovery delay values τ. The signal amplitudes from the data were fitted with a stretch exponential function of the form
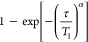
1(with α ranging from 0.7 to 1). The stretch exponential was used to account for a distribution of correlation times, τ_c_, temperature gradients across the sample, and the inherent multiexponential behavior for relaxation of *I* = ^3^/_2_ nuclei.^37−39^ All ^6^Li and ^31^P shifts were referenced to 10 M LiCl in D_2_O and 85% H_3_PO_4_ in water at 0 ppm, respectively.

^6^Li–^6^Li EXSY NMR experiments were performed on an 18.8 T Bruker NEO spectrometer equipped with a 3.2 mm HX MAS probe with the X channel tuned to ω_0_/2π(^6^Li) = 118 MHz and an MAS rate ω_r_/2π of 20 kHz. Experiments were recorded with a π/2 pulse with a duration of 6.25 μs at an rf field amplitude ω_1_/2π(^6^Li) of 40 kHz and measured using the nuclear Overhauser effect spectroscopy (NOESY) pulse sequence, preceded by a presaturation block to reduce the experiment time due to the long ^6^Li SLR time. The resulting pulse sequence was hence (π/2–*d*)_x100_–*d*_1_–π/2–*t*_1_–π/2−τ_m_–π/2–acq, where *d* is a short delay (1 ms), *d*_1_ the recycle delay (400 s) and τ_m_ the varied mixing time.

A ^31^P–^31^P refocused incredible natural abundance double-quantum transfer experiment (INADEQUATE)^40−42^ was performed on a 9.4 T Bruker Avance III HD spectrometer equipped with a 4 mm HXY MAS probe (in double-resonance mode) with the X channel tuned to ω_0_/2π(^31^P) = 162 MHz and an MAS rate ω_r_/2π of 10 kHz. The INADEQUATE was performed with a π/2 pulse with a duration of 3 μs at a rf field amplitude ω_1_/2π(^31^P) of 83 kHz and measured using the refocused INADEQUATE pulse sequence^40^ with a presaturation block due to the extremely long ^31^P SLR time. The resulting pulse sequence was hence (π/2–*d*)_x100_–*d*_1_–π/2−τ–π–τ–π/2–*t*_1_–π/2−τ–π–τ–acq, where *d* is a short delay (1 ms), *d*_1_ the recycle delay (480 s) and τ the refocusing time. τ was optimized for maximum signal intensity, resulting in an evolution period of 6 ms, which is slightly shorter than would be expected for 1/(4J), ∼12.5 ms (using an approximate ^2^*J*_P–P_ of 20 Hz),^43^ due to the magnetization loss during the evolution period from the short ^31^P transverse relaxation time (*T*_2_′ ∼ 5 ms) measured via a spin echo experiment.

### Variable-Temperature NMR Experiments

Variable-temperature ^7^Li NMR experiments were performed with a 4 mm HX high-temperature (HT) MAS probe on a 9.4 T Bruker Avance III HD spectrometer under static conditions with the X channel tuned to ^7^Li at ω_0_/2π(^7^Li) = 156 MHz. The sample was sealed in a glass ampule, and the spectra were recorded with a pulse length of 1.5 μs at a rf field amplitude ω_1_/2π of 83 kHz and referenced to 10 M LiCl in D_2_O at 0 ppm. All ^7^Li one-pulse NMR spectra were obtained with quantitative recycle delays of >5 times the *T*_1_ time constants at each temperature, with *T*_1_ being measured using the saturation recovery pulse sequence as described above. The ^6^Li static *T*_1_ time constant was also recorded in this manner. *T*_1ρ_ time constants were recorded using a spin-lock pulse sequence preceded with a presaturation block to reduce the experiment time and ensure all sites were in a steady state. Hence, the pulse sequence used was of the form (π/2–*d*)_x100_–*d*_1_–π/2–spin lock–acq (where *d* is a short delay (1 ms), *d*_1_ the recycle delay ranging from 90 to 150 s and with the duration of spin-lock *τ* being incremented) at various spin-lock frequencies ω_1_/2π(^7^Li) of 25, 50, and 80 kHz, and the signal amplitudes from the data were fitted to a stretch exponential function of the form
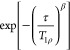
2(with β ranging from 0.4 to 0.8). To measure *T*_1ρ_ time constants at temperatures below 390 K, strong rf pulses much longer than 50 ms would be required, which is beyond the NMR probe capabilities, and their *T*_1ρ_ values were thus not measured. ^7^Li SAE decay curves were recorded using the three-pulse Jeener–Broekaert sequence.^44^ Due to the extremely long ^7^Li longitudinal relaxation time in Li_3_P_5_O_14_, a presaturation block was also used in the collection of the SAE NMR spectra so that the pulse sequence implemented was (π/2–*d*)_x100_–*d*_1_–(π/2)_*y*_–*t*_p_–(π/4)_*x*_–τ_m_–(π/4)_ϕ_–*t*_p_–acq. A short delay *d* of 1 ms, a recycle delay *d*_1_ of 260 s and a π/2 pulse length was 1.8 μs at a rf field amplitude ω_1_/π(^7^Li) of 70 kHz were used. The Jeener–Broekaert sequence generates quadrupolar order^44,45^ to create stimulated echoes that decay with mixing time τ_m_. The short preparation time, *t*_p_, of only 15 μs ensured the formation of a quadrupolar spin-alignment state while simultaneously suppressing the dipolar contributions. A series of 20 echoes were collected with mixing times ranging from 10 μs to 10 s, at three different temperatures (295, 330, and 373 K). The resulting echo decays, *S*_2_(*t*_p_, τ_m_, τ_c_), as a function of τ_m_ were fitted with a single stretched exponential function of the form
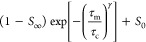
3where *S*_∞_, τ_c_, γ*,* and *S*_0_ are the echo amplitude at τ_m_ = ∞, the correlation time, the stretch exponential, and the echo amplitude at τ_m_ = 0, respectively, with γ ranging from 0.2 to 0.6.

Variable-temperature ^31^P NMR experiments were performed with a 4 mm HX HT MAS probe on a 9.4 T Bruker Avance III HD spectrometer under static conditions with the X channel tuned to ^31^P at ω_0_/2π(^31^P) = 162 MHz. The spectra were recorded with a pulse length of 5 μs at a rf field amplitude ω_1_/2π of 50 kHz; all ^31^P one-pulse NMR spectra were recorded under quantitative recycle delays measured using the same methodology that was used for ^7^Li, and the data were fitted with a stretch exponential (with α ranging from 0.8 to 1). *T*_1ρ_ time constants were measured using a spin-lock pulse sequence with a presaturation block (where the duration of the recycle delay *d*_1_ ranged from 3700 to 4000 s) at a spin-lock frequency ω_1_/2π(^31^P) of 25 kHz, and the data were fit to a stretch exponential (with β ranging from 0.3 to 0.9).

Temperature calibrations were performed with the chemical shift thermometers Pb(NO_3_)_2_ using ^207^Pb NMR^46,47^ and CuI and CuBr using ^63^Cu NMR.^48,49^ The largest errors associated with this method arise from temperature gradients in the sample, which were calculated using the isotropic peak line broadening and range from 5 to 20 K.

### Computational Methods

All density functional theory (DFT) calculations were carried out with the CASTEP (version 20.11) package.^50^ Geometry optimization was performed using plane-wave DFT^51^ with the PBE^52^ exchange-correlation functional and on-the-fly generated ultrasoft pseudopotentials.^53^ The Brillouin zone was sampled at the Γ point using a plane-wave cutoff energy of 850 eV determined by explicit convergence testing with an energy threshold of 1 meV/atom. The electronic energy convergence was set to 1 × 10^–9^ eV/atom. Geometry optimization was carried out using convergence thresholds of 1 × 10^–5^ eV/atom, 3 × 10^–2^ eV/Å, 5 × 10^–2^ GPa, and 1 × 10^–3^ Å for the maximum energy change, maximum force, maximum stress, and maximum displacement, respectively. All NMR parameters were calculated on the optimized geometry using the GIPAW (gauge including projector-augmented waves) approach.^54,55^ The calculations yield absolute shielding tensor **σ** in the crystal frame. According to the Haeberlen convention,^56^ diagonalization of the symmetric part of **σ** gives the three principal components (σ_*xx*_, σ_*yy*_, and σ_*zz*_) such that |σ_*zz*_ – σ_iso_| ≥ |σ_*xx*_ – σ_iso_| ≥ |σ_*yy*_ – σ_iso_|. **σ** is expressed in terms of the isotropic chemical shielding σ_iso,cs_ = ^1^/_3_(σ_*xx*_ + σ_*yy*_ + σ_*zz*_), the anisotropic chemical shielding σ_aniso,cs_ = σ_*zz*_ – ^1^/_2_(σ_*xx*_ + σ_*yy*_), and the asymmetry parameter η = (σ_*yy*_ – σ_*xx*_)/(σ_*zz*_ – σ_iso_). To facilitate the comparison between computational and experimental results, the computed isotropic chemical shielding, σ_iso_, was converted into an isotropic chemical shift, δ_iso,cs_, using the equation δ_iso,cs_ = σ_ref_ + *m*σ_iso_. Anisotropic chemical shift δ_aniso,cs_ is obtained from the computed anistropic chemical shielding using the equation δ_aniso,cs_ = *m*σ_aniso,cs_, where σ_aniso,cs_ is the anisotropic chemical shielding. For ^6^Li, *m* and σ_ref_ were taken from our calculations of Li_2_O, LiOH, and Li_2_CO_3_ and compared with experimental shifts from the literature,^57^ yielding a σ_ref_ of 89.47 ppm and an *m* of −0.998. For ^31^P, the experimentally observed chemical shifts were plotted versus the resulting predicted chemical shieldings, yielding a σ_ref_ of 217.6 ppm and an *m* of −0.777, and the resulting linear relationship was used to optimize the predicted ^31^P chemical shifts (Figure S2). Simulations of the predicted NMR spectra shown are produced with the solid line shape analysis tool “Sola” in Topspin.

## Results and Discussion

### ^31^P MAS NMR

**Figure 2:**
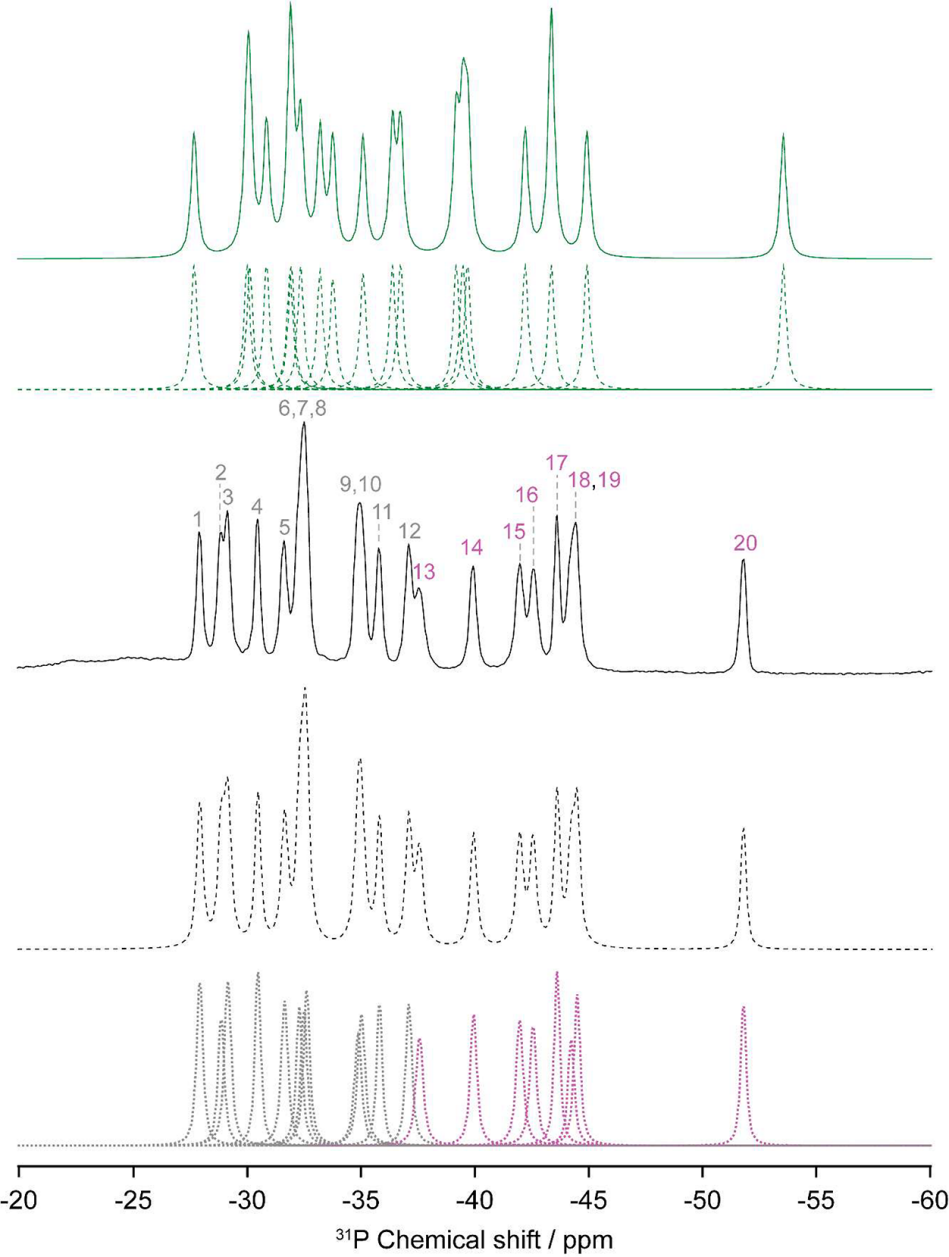
^31^P MAS spectrum of Li_3_P_5_O_14_ with the spectral assignment corresponding to the P sites in Figure 4a. The experimental spectrum (solid black line), total fit (dashed black line), spectral deconvolution (dotted gray and pink lines), and GIPAW-simulated spectrum (green line) are shown. PO_4_^3–^ groups that bridge to two and three other PO_4_^3–^ tetrahedra are colored gray and pink, respectively.

**Figure 3:**
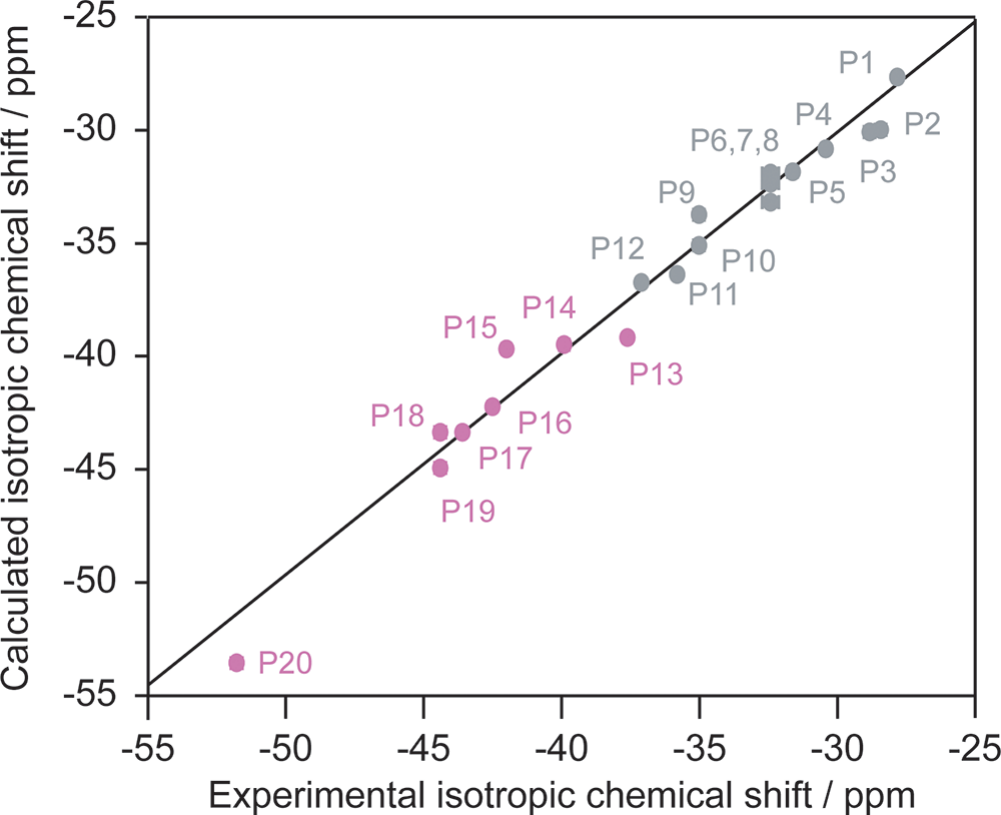
Comparison between the CASTEP-calculated ^31^P isotropic chemical shift and the experimentally observed ^31^P isotropic chemical shift from the MAS NMR spectrum of Li_3_P_5_O_14_ (Figure 2). The points are colored gray and pink for PO_4_^3–^ groups that share corners with two and three other PO_4_^3–^ tetrahedra, respectively. The solid black line corresponds to a linear fit of the data, and a majority of the error bars for the experimental data are within the data point.

^31^P MAS NMR was used to gain insight into the local environment of the PO_4_^3–^ tetrahedra (Figure 1a). The ^31^P MAS NMR spectrum of Li_3_P_5_O_14_ (Figure 2) displays a significant number of narrow resonances spanning 30 ppm and centered at approximately −40 ppm, typical for ultraphosphate rings.^58^ From the experimental spectrum, at least 16 resonances can be discerned. In Li_3_P_5_O_14_, there are 16 formula units per unit cell with 20 crystallographically distinct, equally populated phosphorus atoms in the asymmetric unit in the crystal structure (Figure 1). However, a number of the resonances overlap due to the rather similar chemical environments of the PO_4_^3–^ tetrahedra, challenging the assignment of the ^31^P resonances. Through the deconvolution of the ^31^P resonances, integration of the individual resonances (Table 1), and the fitting of the spinning side bands in the MAS NMR spectrum, 20 resonances can be deciphered as fully expected from the crystallography data. Moreover, the fitting of the spinning side bands of the ^31^P spectrum allows for the extraction of CSA values (Figure S3). Importantly, the experimental data reveal that the eight resonances with the lowest isotropic chemical shifts δ_iso,cs_ (−52 to −37 ppm) have significantly lower CSAs by approximately 30-40 ppm than the remaining 12 resonances at higher δ_iso,cs_ values (greater than −37 ppm). The full assignment of the one-dimensional ^31^P MAS NMR spectrum is challenged by the large number of resonances, degree of overlap, and similar chemical environments of the phosphate groups. DFT calculations carried out with GIPAW in CASTEP yielded computed absolute chemical shielding tensors that can be converted into δ_iso,cs_ (Table 1 and Figure 3) that can be used to preliminarily assign the ^31^P MAS NMR spectrum.

**Table 1:** Summary of the Assignment of the ^31^P MAS NMR Spectrum of Li_3_P_5_O_14_, the NMR Parameters Obtained Experimentally (lightface text) and Calculated (boldface text) Using the GIPAW Method Implemented in CASTEP as Well as the SLR Times Obtained through the Saturation Recovery Pulse Sequence

no. of bridging PO_4_^3–^ tetrahedra	assignment	δ_iso,cs_ (ppm)^a^	δ_aniso,cs_ (ppm)^b^	η^c^	*T*_1_ (s)
2	P1	–27.9/**–27.7**	–167.2/**–150.0**	0.38/**0.38**	1580 ± 256
	P2	–28.5/**–30.0**	–167.1/**–154.4**	0.34/**0.27**	1474 ± 177
	P3	–28.9/**–30.1**	–166.3/**–145.5**	0.25/**0.30**	1496 ± 169
	P4	–30.5/**–30.8**	–161.4/**–141.4**	0.43/**0.36**	1380 ± 160
	P5	–31.7/**–31.9**	–174.6/**–136.8**	0.29/**0.38**	1553 ± 126
	P6	–32.5/**–31.9**	–166.5/**–146.7**	0.31/**0.35**	1562 ± 77
	P7	–32.5**/–32.4**	–166.5/**–156.8**	0.32/**0.26**	1562 ± 77
	P8	–32.5/**–33.2**	–166.5/**–154.1**	0.32/**0.32**	1562 ± 77
	P9	–35.1/**–33.7**	–172.6/**–154.8**	0.31/**0.32**	1652 ± 79
	P10	–35.1/**–35.1**	–172.6/**–161.2**	0.34/**0.29**	1652 ± 79
	P11	–35.9/**–36.4**	–177.7/**–120.4**	0.24/**0.04**	1688 ± 181
	P12	–37.2/**–36.7**	–174.7/**–162.6**	0.34/**0.28**	1663 ± 86
3	P13	–37.7/**–39.2**	–134.1/**–109.9**	0.24/**0.20**	1688 ± 98
	P14	–40.0/**–39.5**	–126.2/**–164.5**	0.23/**0.27**	1731 ± 68
	P15	–42.1/**–39.7**	–122.3/**–100.1**	0.18/**0.14**	1603 ± 38
	P16	–42.6/**–42.2**	–138.1/**–114.2**	0.05/**0.15**	1590 ± 87
	P17	–43.7/**–43.4**	–134.2/**–121.2**	0.23/**0.15**	1613 ± 63
	P18	–44.5/**–43.4**	–136.4/**–102.1**	0.10/**0.03**	1758 ± 145
	P19	–44.5/**–44.9**	–127.2/**–120.4**	0.08/**0.04**	1758 ± 145
	P20	–51.9/**–53.5**	–135.0/**–117.7**	0.20/**0.14**	1938 ± 119

a Experimental and computed isotropic chemical shifts were obtained via the deconvolution of the ^31^P NMR spectra in Figure 2 and from the computed σ_iso_ values via the expression δ_iso,cs_ = σ_ref_ + *m*σ_iso_, respectively.

b Experimental δ_aniso,cs_ values were obtained through the fitting of the spinning side band manifold of the ^31^P NMR spectrum in Figure S3. Computed δ_aniso,cs_ values were obtained via the calculated σ_aniso,cs_ Haeberlen convention, such that σ_aniso,cs_ = σ_*zz*_ – ^1^/_2_(σ_*xx*_ + σ_*yy*_) and δ_aniso,cs_ = *m*σ_aniso,cs_.

c Experimental η values were obtained through the fitting of the spinning side band manifold of the ^31^P NMR spectrum in Figure S4. Computed η values were obtained via the Haeberlen convention, such that η = (σ_*yy*_ – σ_*xx*_)/(σ_*zz*_ – σ_iso_).

**Figure 4:**
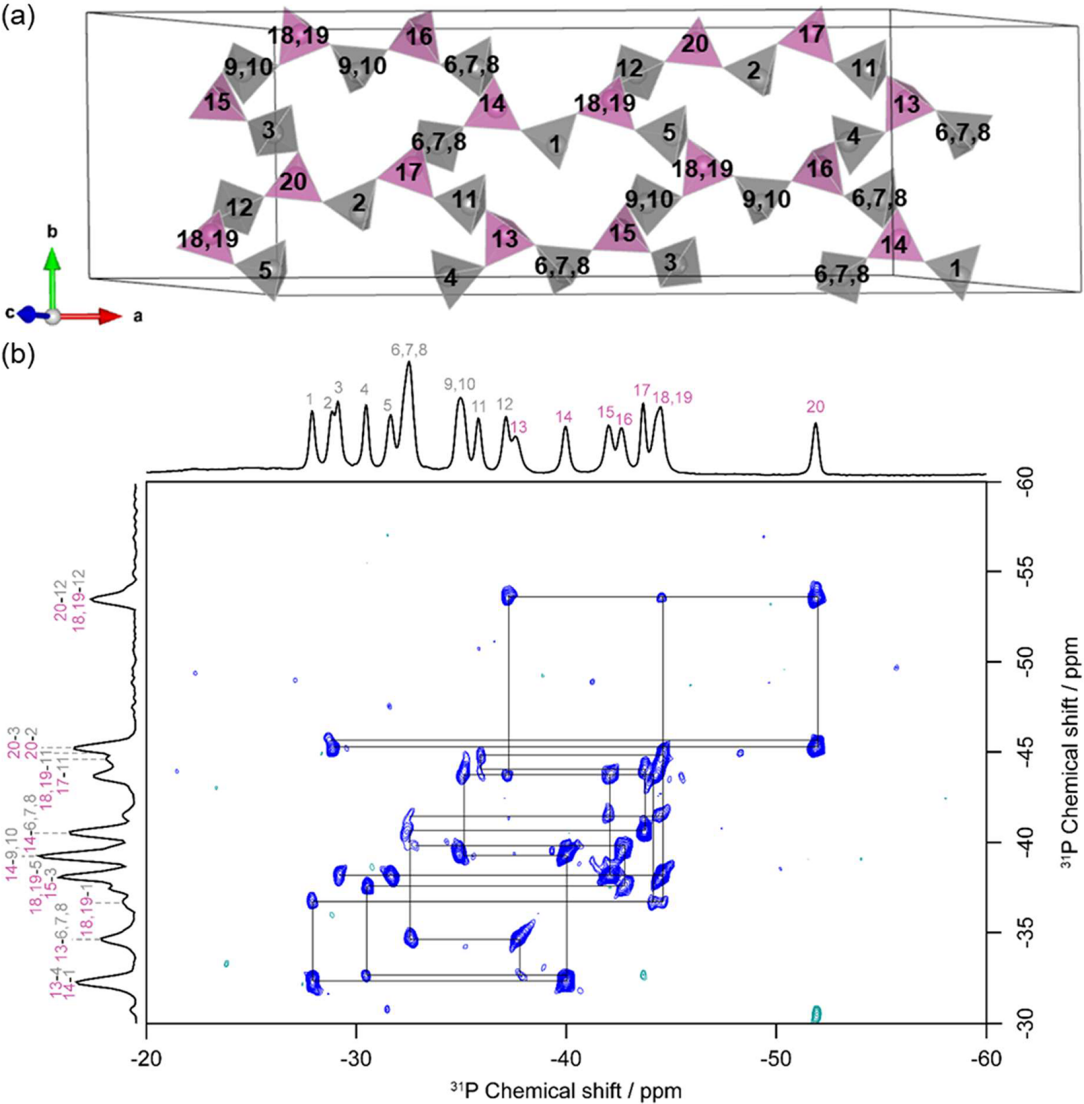
(a) Crystal structure of Li_3_P_5_O_14_ displaying the central P_20_O_56_^12–^ ultraphosphate layer in the unit cell and the assignment of the different P sites on the basis of the ^31^P MAS NMR and INADEQUATE spectra. PO_4_^3–^ groups are color coded according to their type, where units that bridge to three other tetrahedra are colored pink and PO_4_^3–^ units that bridge to two other tetrahedra are colored gray. O atoms have been omitted for clarity. Only half of the 16 formula units per unit cell in Li_3_P_5_O_14_ are shown for simplicity. Note the labelling of the crystallographic P sites obtained here through ^31^P NMR, differs from the labelling from previously reported diffraction data (ICSD 114286).^20^ A comparison of the two labelling systems is shown in Table S1. (b) 2D ^31^P-^31^P refocused INADEQUATE NMR spectrum of Li_3_P_5_O_14_ showing the observable correlations with black lines. Selected correlations are highlighted on the indirect dimension. The spectral window focuses on the isotropic region; however, some correlations are more easily seen in the region of the first spinning side band (Figure S5). A stack plot of a selection of one-dimensional slices at a range of double-quantum frequencies is shown in Figure S6.

Upon close inspection of the crystal structure of the ultraphosphate layer in Li_3_P_5_O_14_, two types of rings repeat along the *b*-axis (Figure 4a). The 20 PO_4_^3–^ tetrahedra in these rings can be divided into two categories, eight PO_4_^3–^ groups that have three bridging O atoms to other PO_4_^3–^ groups (pink in Figure 4a) and 12 phosphate groups that have two bridging O atoms to other phosphate groups. This observation signifies that the eight resonances with the lowest isotropic chemical shift and chemical shift anisotropy correspond to the eight P sites that exist in PO_4_^3–^ tetrahedra that bridge to three other PO_4_^3–^ groups, while the remaining 12 P resonances correspond to the P atoms with two bridging O atoms to other PO_4_^3–^ groups.

The ^31^P SLR times for the various sites in Li_3_P_5_O_14_ are extremely long [∼1600 s (Table 1)], as commonly observed for ^31^P nuclei,^59^ while also further confirming the crystallinity of the sample. The long relaxation times are postulated to be due to the lack of efficient pathways for relaxation with any nearby NMR active nuclei near the P atoms. These are ^17^O atoms that exist in very low natural abundance (0.038%), and any dipolar relaxation or relaxation from dipolar/scalar coupling of the quadrupolar coupling to ^17^O nuclei will thus be minimal. The primary mechanisms for ^31^P SLR will likely come from CSA and homonuclear dipolar coupling to neighboring ^31^P nuclei, as well as heteronuclear dipolar relaxation and relaxation arising from the nearest quadrupolar ^7^Li nuclei. However, the magnitude of these dipolar interactions decreases as the inverse cube of the interatomic distances which are large (about 2.9 Å for P–P distances given the P–O–P units and 3.2 Å for P–Li distances given the ultraphosphate/Li polyhedral layered structure (Figure 1c)); hence, the most prominent relaxation mechanism will likely be CSA. At recycle delays (<20 s) much shorter than the *T*_1_ times of Li_3_P_5_O_14_, a broad peak emerges at approximately −25 ppm, likely corresponding to an amorphous phase (∼12% from integral) that was not detected through diffraction measurements (Figure S4).

To experimentally and unambiguously assign the large number of ^31^P resonances in Figure 2, a refocused NMR INADEQUATE spectrum was collected; this experiment probes through-bond ^2^*J*_P-P_ scalar couplings and is a valuable technique for interpreting the ^31^P-O-^31^P connectivity in Li_3_P_5_O_14_ (Figure 4b). The correlations in the *J* coupling-based ^31^P-^31^P refocused INADEQUATE are due to P atoms that are two bonds away from one another and yield a resonance at the double-quantum frequency in the indirect dimension that is the sum of their individual frequencies in the single-quantum dimension (Ω_PaPb_ = ω_Pa_ + ω_Pb_). The proposed assignment of the numerous ^31^P MAS NMR spectra was completed (Figure 4a) using a combination of the observed CSA allowing for the identification of the PO_4_^3–^ units that share corners with three other tetrahedra along with the INADEQUATE NMR spectra, and the resulting central ultraphosphate layer can be identified. For example, site P12 (−37.2 ppm) shares corners with two other phosphate groups, P18/19 (−44.5 ppm) and P20 (−51.9 ppm), which in turn share corners with three other tetrahedra; for P20, these three are namely P2 (−28.5 ppm), P3 (−28.9 ppm), and P12 (−37.2 ppm). A stack plot of traces of the observed correlations extracted from the 2D INADEQUATE spectrum is shown in Figure S6. These traces display a trend of greater signal intensity for the resonance corresponding to the P sites with a higher degree of connectivity. Generally, it would be expected that both signals in a correlation from an INADEQUATE spectrum should be of equal amplitude as the 1D spectrum; however, this intrinsic asymmetry can be explained due to the large difference in CSA between the two types of P sites in Li_3_P_5_O_14_. As shown in Table 1, the P sites with greater connectivity to other PO_4_^3–^ groups possess lower CSA and hence fewer pathways for relaxation; therefore, the *T*_2_ relaxation time for these sites will be longer and less magnetization will be lost during the refocusing time, increasing signal intensity.

### ^6^Li MAS NMR

**Figure 5:**
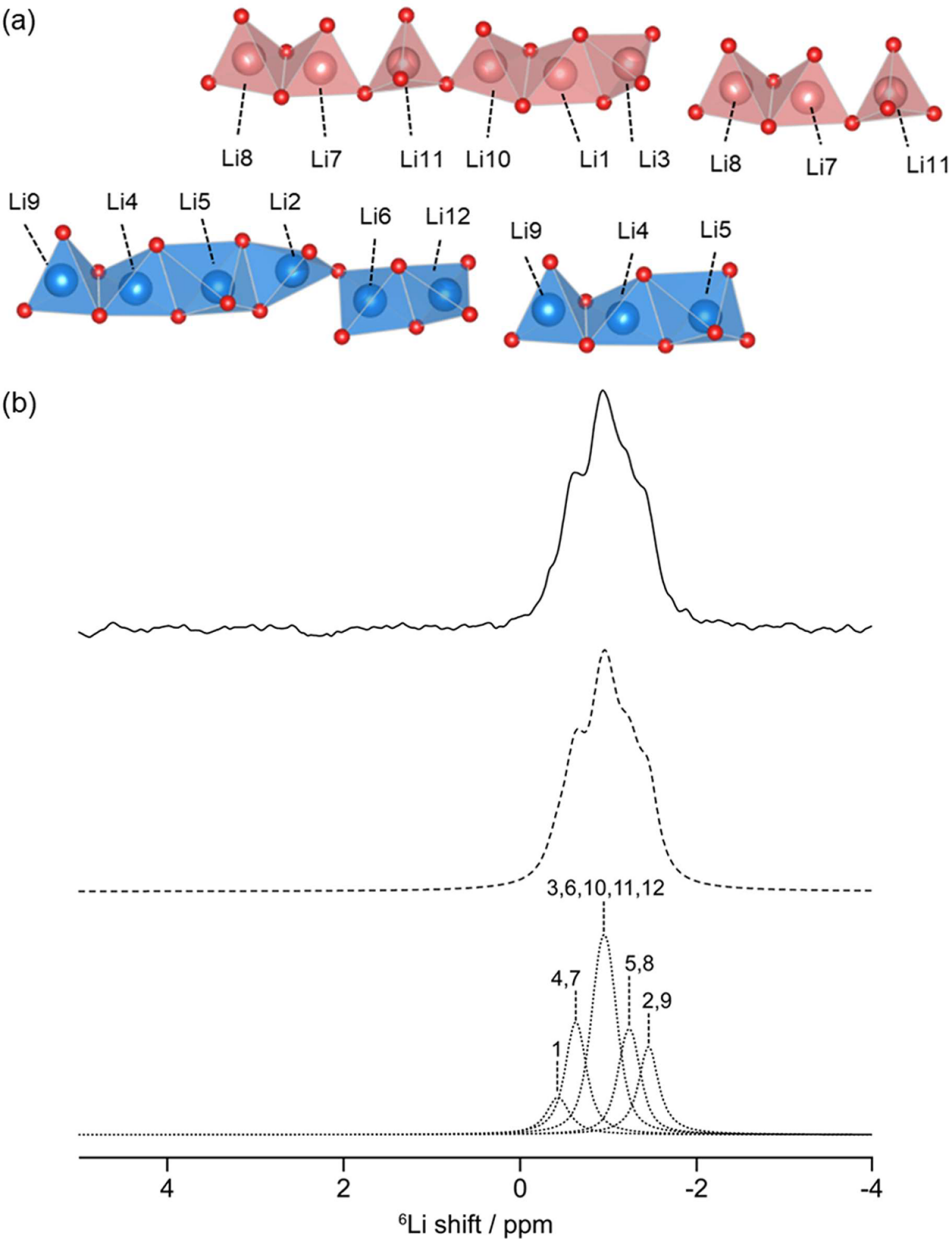
(a) Crystal structure of the Li polyhedral layers in Li_3_P_5_O_14_ displaying the two types of Li_6_O_16_^26–^ chains, type A (red) and type B (blue), with different connection modes along with two distinct vacant tetrahedral sites at the terminating ends. Li site labelling is consistent with the previously reported crystal structure (ICSD 114286)^20^ (b) Room-temperature ^6^Li MAS spectrum of Li_3_P_5_O_14_ obtained at 9.4 T along with the spectral assignment based on the difference in the shifts of the various sites from DFT calculations. The experimental spectrum (solid black line), total fit (dashed black line), and spectral deconvolution (dotted lines) are shown.

Further information about the local arrangement of the atoms in Li_3_P_5_O_14_ can be obtained through ^6^Li MAS NMR (Figure 5). The ^6^Li MAS NMR spectrum of Li_3_P_5_O_14_ displays several overlapping resonances centered at approximately −1 ppm, from which five resonances can be deconvoluted integrating to 1:2.2:4.6:2:1.9 (a comparison of the residual spectra for deconvoluting with four or five resonances is shown in Figure S7). Twelve resonances of equal intensity are expected from the crystal structure and correspond well with the sum of the integrations obtained from NMR. Due to the large number of expected resonances and the complex nature of the Li_6_O_16_^26–^ chains, GIPAW calculations of Li_3_P_5_O_14_ were utilized for the assignment of these resonances in the two Li_6_O_16_^26–^ chains (Figure 5a). The observed shifts of the ^6^Li resonances in the simulated spectrum are significantly different from those observed in the room-temperature MAS spectrum; however, this is to be expected as DFT calculations are performed assuming a temperature of 0 K and the shift in Li_3_P_5_O_14_ is strongly dependent on temperature (Figure S8), likely capturing lithium ion motion. Therefore, the assignment of the resonances in Figure 5b was based on the integrations in the MAS NMR spectrum and the ordering of the calculated shifts obtained from lowest to highest obtained from the DFT calculations (Table S2).

Upon closer inspection of the Li polyhedra and the assignment of the shifts using GIPAW calculations, we observe that the shielding is related to the number of O atoms in the Li polyhedra that are bonded to three other atoms. For example, in the LiO_4_ tetrahedra for Li1 and Li7, all of the O atoms in this tetrahedra are shared between three atoms, leading to an increase in electron density around the ^6^Li nucleus and a smaller shift. These two Li sites also share edges with adjacent LiO_4_ tetrahedra, leading to a decrease in the Li–Li interatomic distance (∼ 2.5 Å) and additional shielding of this site. The resonances associated with the remaining Li sites also follow this trend, with the observed shift increasing as the number of atoms in the Li polyhedra that have three bonds decreases. Notably, our assignment seems to disagree with the semiempirical correlations relating the lithium coordination environment and ^6^Li NMR shifts,^60^ with the distorted square pyramid site appearing at a shift higher than those of a number of the tetrahedral LiO_4_ sites. However, this observation is not entirely unexpected, as the additional shielding from one additional O atom will be minimal. The extremely narrow chemical shift range of ^6^Li means the assignment of a number of Li resonances based on coordination number is more complex than this empirical correlation and computational calculations appear to be more reliable.

### ^6^Li Variable-Temperature MAS NMR

**Figure 6:**
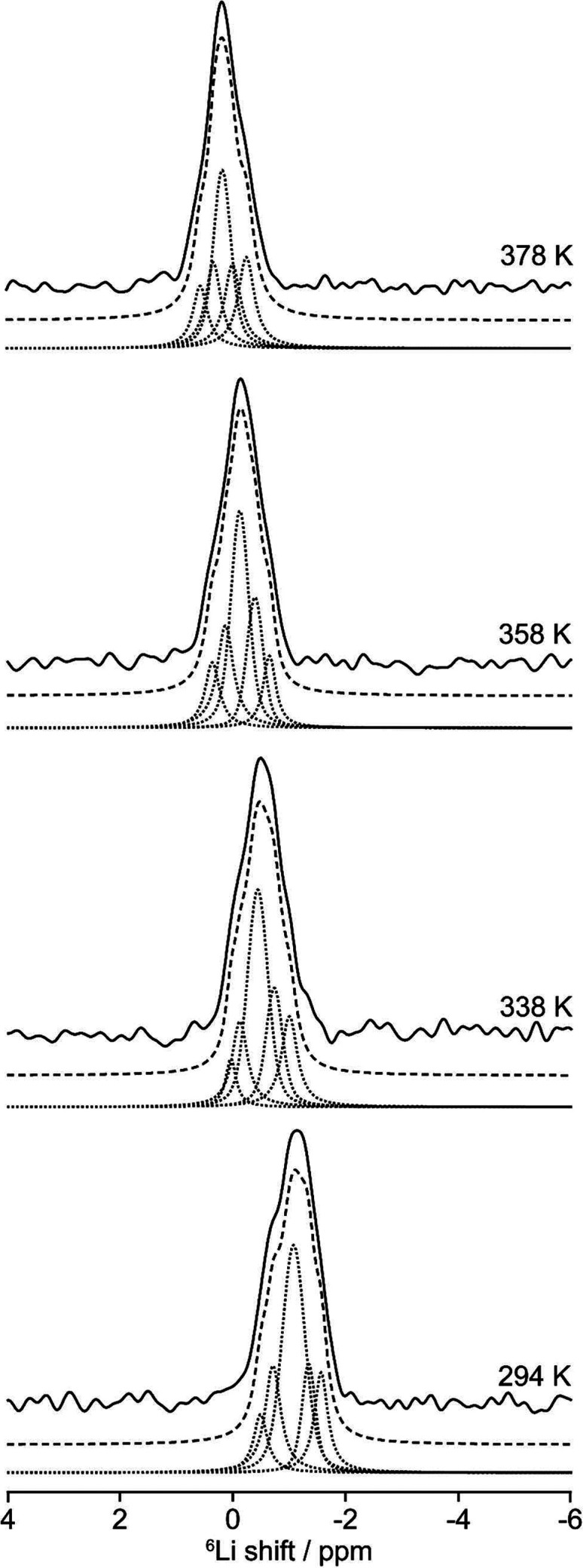
^6^Li MAS NMR spectra of Li_3_P_5_O_14_ measured at 20 T as a function of the temperature. The experimental spectrum (solid black lines), total fit (dashed black lines), and spectral deconvolution (dotted lines) are shown.

To gain insight into the microscopic Li ion mobility in Li_3_P_5_O_14_, ^6^Li variable-temperature MAS NMR was recorded (Figure 6). The resulting spectra display a dependence of the observed shift with temperature, wherein the shifts increase with an increase in temperature (Figure S8). A reduction in resolution at 20 T is observed in the ^6^Li MAS NMR spectrum compared to 9.4 T (Figures 5b and 6), which is due to increased inhomogeneous broadening arising from a distribution of shifts at higher external magnetic field strengths due to the larger chemical shift dispersion; e.g., the five sites that appear at −1 ppm in the room-temperature NMR data are likely only partially resolved at 20 T, and this is reflected in an apparent increase in the overall line width, reducing resolution. The fitting of the 20 T data is therefore based on the deconvolution of the low-field data with the signal-to-noise ratio (SNR) consistent with the 20 T data; the increased sensitivity of the high-field data is beneficial in this case due to the long ^6^Li relaxation times, which otherwise would challenge the SNR. The five separate ^6^Li resonances obtained from the 9.4 T data can be observed in each of the variable-temperature MAS spectra at 20 T, the intensities of which vary very little over the temperature range explored (between room temperature and 378 K).

Attempts to obtain site specific Li ion jump rate τ^–1^ values through homonuclear ^6^Li-^6^Li EXSY NMR (Figure S9) at 18.8 T were made. Exchange is observed experimentally in the form of off-diagonal cross peaks in the 2D EXSY spectra at the corresponding shifts at a rate governed by mixing time τ_m_. As τ_m_ increases, the intensity of the corresponding cross peaks increases (ignoring relaxation effects) at a rate proportional to the Li ion exchange rate. By performing these experiments at a range of τ_m_ values, we can fit the build-up of the cross peaks to obtain site specific Li ion τ^–1^ values. Due to the significant degree of overlap in the ^6^Li NMR resonances and cross peaks, unfortunately, no reliable values could be obtained. However, qualitatively, one can see that at a τ_m_ value of 5 s, cross peaks are clearly visible between almost all resonances, indicating that Li mobility occurs via each of the individual Li sites. Upon close inspection of the ^6^Li-^6^Li EXSY NMR spectra, cross peaks occur between site Li1 (−1 ppm) and sites Li5 and Li8 (−1.2 ppm) as well as Li2 and Li9 (−1.4 ppm). Given that Li1 resides in the A type Li_6_O_16_^26–^ chain while Li2, Li5, and Li9 reside at adjacent positions in the B type chain, this result suggests that intralayer (in the *a-b* plane) Li ion mobility is feasible. Interestingly, Li5 and Li2 are positioned above and below Li1, respectively (along the *c*-axis), meaning that for Li ion diffusion to take place between these sites, the ions must pass through the 12-membered P_12_O_36_^12–^ rings.

### Relaxation Measurements

The Li ion mobility in Li_3_P_5_O_14_ was probed at a range of time scales through SLR rate constants in the laboratory frame *T*_1_^–1^ and the rotating frame *T*_1ρ_^–1^, which provide information about the ion dynamics on the scales of megahertz and kilohertz, respectively. The motion of atoms or functional groups causes a random change in the local magnetic fields, which leads to relaxation, quantitative information about the ion mobility process being contained in these microscopic changing fields. τ_c_ describes the time scale of these fluctuations, and Bloembergen-Purcell-Pound (BPP) theory postulates that the main factor influencing the reorientation of the local magnetic fields is the increased mobility of ^7^Li nuclei with increasing temperatures. The spectral density function *J*(ω_0_) quantifies the motion at Larmor frequency ω_0_:^61,62^
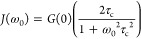
4where *G*(0) is the value of the correlation function at time zero and is equal to the mean square of the local magnetic fields. Because the primary factor affecting the reorientation of the local magnetic fields is, in this work, the increased mobility of ^7^Li nuclei, the temperature-dependent changes in τ_c_ are solely induced by the diffusion and follow an Arrhenius relation of the type
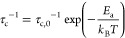
5where τ_c,0_^–1^ is the Arrhenius pre-exponential factor, *E*_a_ the activation energy, *T* the temperature, and *k*_B_ the Boltzmann constant. To gather information about the activation energy, conductivity, and dimensionality of the Li diffusion processes, the temperature dependence of the ^7^Li SLR rate constants under static conditions was collected and exploited.

**Figure 7:**
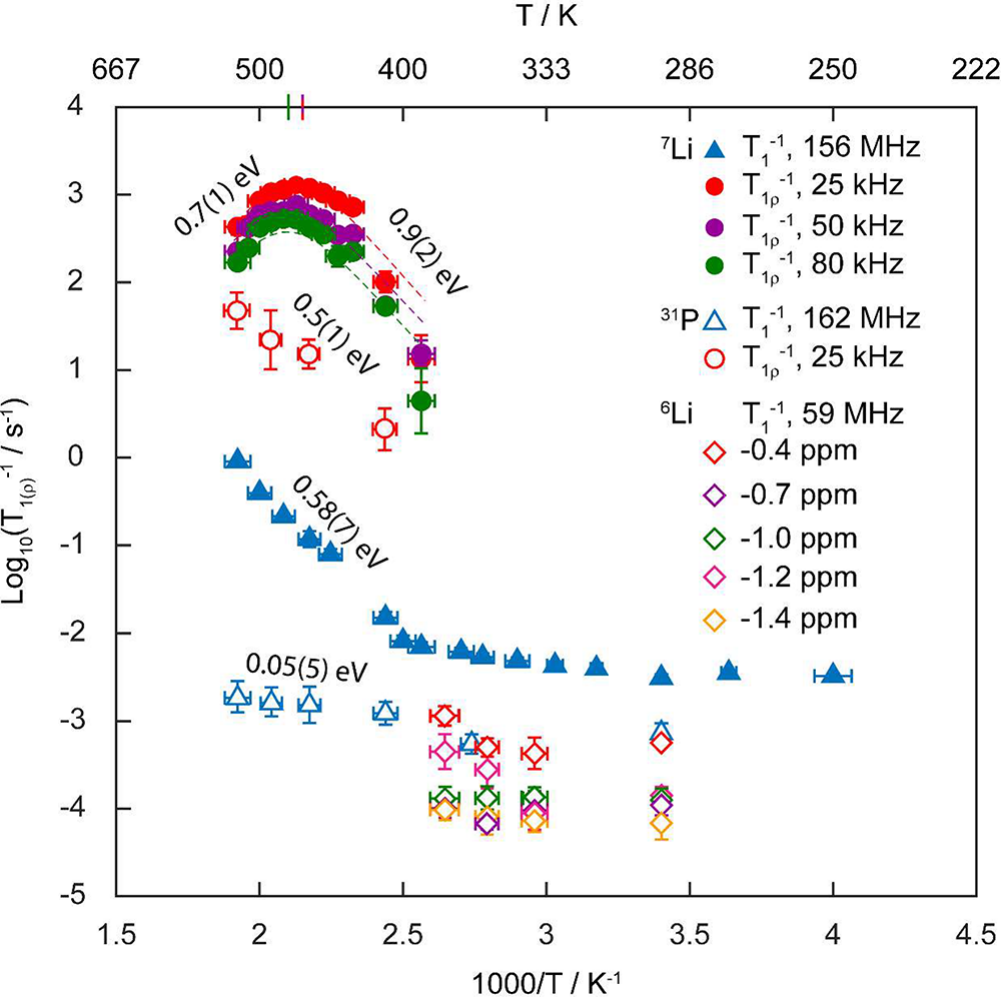
Arrhenius plots of ^6,7^Li and ^31^P NMR SLR rate constants in the laboratory (*T*_1_^–1^) at ω_0_/2π = 59, 156, and 162 MHz, respectively (blue triangles and colored diamonds for ^7^Li/^31^P and ^6^Li, respectively), and rotating frame (*T*_1ρ_^–1^) at ω_1_/2π = 25 kHz (red circles), 50 kHz (purple circles), and 80 kHz (green circles) for ^7^Li and 25 kHz for ^31^P. ^7^Li SLR rate constants are denoted with filled shapes, while ^6^Li and ^31^P SLR rate constants are represented by empty shapes. The colored ticks on the temperature scale represent the position of the ^7^Li *T*_1ρ_^–1^ maxima (note that the maxima for ω_1_/2π values of 25 and 50 kHz occur at the same temperature within the temperature accuracy and gradient as given in the Experimental Section, and a tick alternating in red and purple is used in this case). Colored dashed lines outline the fitting of the experimental data to eq 8. *T*_1ρ_ time constants at temperatures below 390 K could not be collected, as values exceed 50 ms and are beyond the NMR probe capabilities. A magnified view of the region covering the ^7^Li *T*_1ρ_^–1^ maxima is shown in Figure S10.

The ^7^Li SLR *T*_1_^–1^ rate constants for Li_3_P_5_O_14_ are largely independent temperature in the range of 250-330 K and vary from 3 to 4 × 10^–3^ s^–1^. This is to be expected in this regime where the SLR rates are not induced by diffusion due to the absence of (translational) Li ion mobility.^63^ When Li_3_P_5_O_14_ is heated from 330 to 520 K, *T*_1_^–1^ increases from 4 × 10^–3^ to 0.9 s^–1^, which follows Arrhenius behavior, and hence, an *E*_a_ of 0.58(7) eV can be extracted (Figure 7). The increase in the SLR *T*_1_^–1^ rate constants with temperature implies data in the low-temperature flank of the SLR rate constants, which are indicative of short-range motional processes where τ_c_ ≪ ω_0_ and correspond to the slow motion regime where the motion of spins between sites is restricted and does not exchange between sites within a single precession of ω_0_.^61,64,65^

The ^7^Li SLR rates recorded in the rotating frame were obtained at three different spin-lock frequencies (ω_1_/2π) of 25, 50, and 80 kHz (Figure 7). The rates initially increase with temperature (the low-temperature flank characterizes local short-range motional processes) with an activation barrier of 0.9(2) eV. Upon further heating, the SLR *T*_1ρ_^–1^ rate constants reach a maximum value (at 470 K for ω_1_/2π = 25 and 50 kHz and 480 K for ω_1_/2π = 80 kHz) before decreasing with with an *E*_a_ of 0.7(1) eV; this high-temperature flank contains information for long-range Li ion mobility.

At the temperatures of the *T*_1ρ_^–1^ maxima, the Li^+^ τ_c_^–1^ values are on the order of spin-lock probe frequency ω_1_ and satisfy the relationship^64^
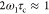
6τ_c_^–1^ values on the order of 3.2 × 10^5^ to 1.0 × 10^6^ s^–1^ are therefore obtained at 470 and 480 K (the experimentally collected *T*_1ρ_^–1^ rates for ω_1_/2π values of 25 and 50 kHz have maximum values at the same temperature) for Li_3_P_5_O_14_.

The SLR values can be further parametrized using the following expression to extract τ_c_ from *T*_1_^–1^ rates:
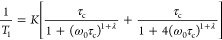
7and from *T*_1ρ_^–1^
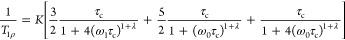
8where *K* is the local fluctuating magnetic field term in these expressions, which depends on the relaxation mechanism, and λ in this case is the exponent of the underlying exponential correlation function and ranges from 0 to 1. A λ of 1 describes a Lorentizian-shaped *J*(ω) and is ascribed to uncorrelated three-dimensional motion, and a λ of <1 accounts for asymmetry in *J*(ω) and often indicates correlated motions when found on the low-temperature flank.

In the case of *S* = ^3^/_2_ nuclei such as ^7^Li, if the homonuclear dipolar relaxation is the dominant relaxation mechanism, *K* is proportional to the square of the dipolar coupling constant and is given by^23^
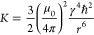
9where μ_0_ is the permeability of free space, ℏ the reduced Planck’s constant, γ the gyromagnetic ratio of the nuclear spins, and *r* is the interatomic distance between the two nuclear spins. In the case of quadrupolar relaxation being the dominant relaxation mechanism, *K* is proportional to the quadrupolar tensor parameters and expressed as
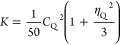
10where *C*_Q_ and *η*_Q_ are the quadrupolar coupling constant and the asymmetry parameter, respectively. It is possible to postulate a dominant relaxation mechanism through ^6,7^Li NMR and is best obtained from comparing ^6^Li and ^7^Li *T*_1_ time constants under static conditions.^66^

Given the power law of 4 and quadratic dependencies of *T*_1_^–1^ on *γ* and quadrupolar moment *Q* in the dipolar and quadrupolar relaxation mechanisms, respectively, a ratio of
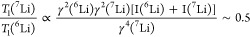
11is expected in the case of dipolar relaxation, while a ratio of
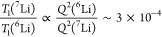
12is anticipated for a quadrupolar relaxation mechanism. In the case of Li_3_P_5_O_14_, experimental *T*_1_(^7^Li) and *T*_1_(^6^Li) values are 3.0(2) × 10^2^ and 1.5(3) × 10^4^ s, respectively, yielding a ratio of 2(1) × 10^–2^ at room temperature under static conditions, suggesting that the overall SLR is caused by either cross relaxation processes or a combination of the two mechanisms.

Upon combination of eqs 5 and 8, an expression of the SLR rate in the rotating frame *T*_1ρ_^–1^ depending on *T* can be extracted to determine the parameters *K*, τ_c,0_^–1^, *E*_a_, and λ. The corresponding fits to the experimental data are shown in Figure 7 with the fitting parameters summarized in Table 2.

**Table 2:** Summary of the Parameters Obtained from ^7^Li *T*_1ρ_^–1^ Relaxation Measurements via the Experimental *T*_1ρ_^–1^ Maxima (eq 6) and through Fitting the Data in Figure 7 with eq 8^a^

ω_1_/2π (kHz)	method	*K* (Hz^2^)	τ_c,0_^–1^ (s^–1^)	*E*_a_ (eV)	λ
25	maxima	6(1) × 10^8^	9.0 × 10^12^	0.67(5)	0.94
	simulation	5.4(3) × 10^8^			
50	maxima	7(1) × 10^8^	2.3 × 10^12^	0.60(6)	0.99
	simulation	6.4(3) × 10^8^			
80	maxima	8(1) × 10^8^	4.8 × 10^11^	0.53(5)	1
	simulation	8.0(4) × 10^8^			
average	maxima	7(1) × 10^8^	3.9 × 10^12^	0.60(6)	0.98
	simulation	6.6(3) × 10^8^			

a Values of *K* are extracted from eqs 6 and 8 and values of τ_c,0_^–1^, *E*_a_, and λ are obtained from the fits to the data in Figure 7.

At the ^7^Li *T*_1ρ_^–1^ maxima, substituting eq 6 into eq 8 enables experimental determination of *K*, and a value of 7(1) × 10^8^ Hz^2^, averaged over the three consistent values of *K* for the three spin-lock frequencies, is extracted for Li_3_P_5_O_14_, which agrees well with the average value of 6.6(3) × 10^8^ Hz^2^ obtained from the fitting of the experimental data in Figure 7. This is to be expected as the two methods are models of the same expression (eq 8); however, the method used from the experimental maxima will likely be slightly less accurate, as the value of *T*_1ρ_^–1^ obtained experimentally may not be the maximum, and it is unlikely that the exact temperature chosen to record this data was the optimum temperature to achieve the highest value of *T*_1ρ_^–1^. This value of *K* can then be used to convert experimental *T*_1ρ_^–1^ values into τ_c_ estimates at each temperature using eq 8 (Figure S12) and allows access to NMR-related τ_c_^–1^ values at all temperatures.

SLR *T*_1ρ_^–1^ rate constants at different frequencies also provide information about the dimensionality of the Li^+^ diffusion process, and for diffusion-induced rates in solids, the high-temperature limits of spectral density function *J*(ω_1_) have the following frequency dependence on (τ_c_/ω_1_)^0.5^, τ_c_ ln(1/ω_1_τ_c_), and τ_c_ for 1D, 2D, and 3D diffusion processes, respectively.^35,36^ The *T*_1ρ_^–1^ rate constants on the high-temperature flank of Li_3_P_5_O_14_ are independent of spin-lock frequency ω_1_/2π (Figure 7 and Figure S10), strongly demonstrating experimentally the presence of 3D Li ion mobility in this material, which is in good agreement with the BVS (bond valence sum)^67^ mapping previously reported and arising from DFT data.^20^ This 3D pathway is believed to occur via both an intralayer (transport in the Li polyhedral layer in the *a-b* plane) and an interlayer (transport between two adjacent Li polyhedral layers with *c*-direction connectivity). The ordered Li_6_O_16_^26–^ chains, shown in Figure 5a along with the vacant tetrahedral sites, form a possible intralayer lithium diffusion pathway in the Li polyhedral layer. The intralayer migration could occur either by a hopping mechanism of Li ions along the Li_6_O_16_^26–^ chains or by hopping between the two types of Li_6_O_16_^26–^ chains, where the local jumps between the two types of Li_6_O_16_^26–^ chains involved are between the two tetrahedral vacancies and adjacent LiO_4_ tetrahedra or the distorted square pyramid site. This information, coupled with the ^6^Li-^6^Li EXSY NMR data (Figure S9), indicates that Li1 likely migrates between Li_6_O_16_^26–^ chains, via intralayer (in the *a-b* plane) and interlayer (along the *c*-axis) mechanisms, providing a likely pathway for long-range 3D ion motion, experimentally verifying the computationally predicted Li ion pathway in Li_3_P_5_O_14_. This potential interlayer Li migration pathway occurs between the 12-membered P_12_O_36_^12–^ rings that provide a window that mobile Li ions can traverse.

The ^31^P SLR rates recorded in the laboratory frame (∼10^–3^ s^–1^) remain constant over the observed temperature range (empty blue triangle, Figure 7), suggesting PO_4_^3–^ group rotation occurs on a time scale much less than the ^31^P Larmor frequency (ω_0_/2π = 162 MHz). This conclusion is further reinforced by the static ^31^P NMR data (Figure S11) in which the resonances are unchanged across a range of temperatures, indicating phosphate reorientation occurs on a time scale less than the static powder pattern (∼20 kHz from the lowest ^31^P CSA value of 122 ppm at this field, Table 1 and Figure S4). The SLR rates in the rotating frame, at a spin-lock frequency of 25 kHz, increase with an activation barrier of 0.5(1) eV. However, no *T*_1ρ_^–1^ maximum is observed, further indicating that phosphate rotation occurs at a rate ω_1_/2π of <25 kHz. Importantly, the activation barriers observed on the low-temperature flanks of the ^7^Li and ^31^P BPP curves are significantly different [0.5(1) eV vs 0.9(2) eV], and the absence of a *T*_1ρ_^–1^ maximum for the ^31^P SLR rates while maxima are observed for ^7^Li at 470 and 480 K are both indicative of ^7^Li translational ion mobility likely not being correlated with PO_4_^3–^ rotation. This is in sharp contrast with other fast Li^+^ conductors such as Li_6_PS_5_X (X = Cl, Br, or I),^68,69^ where correlated motion between rotational jumps of the PS_4_^3–^ units and Li^+^ transport has been observed from ^31^P and ^7^Li BBP curves having the same *E*_a_ as well as *T*_1ρ_^–1^ maximum position. One possible explanation for this is that in materials such as Li_6_PS_5_X the P subunits are isolated and have greater freedom to rotate, while in Li_3_P_5_O_14_, which adopts the ultraphosphate structure, the PO_4_^3–^ units share corners and have less freedom to rotate.

**Figure 8:**
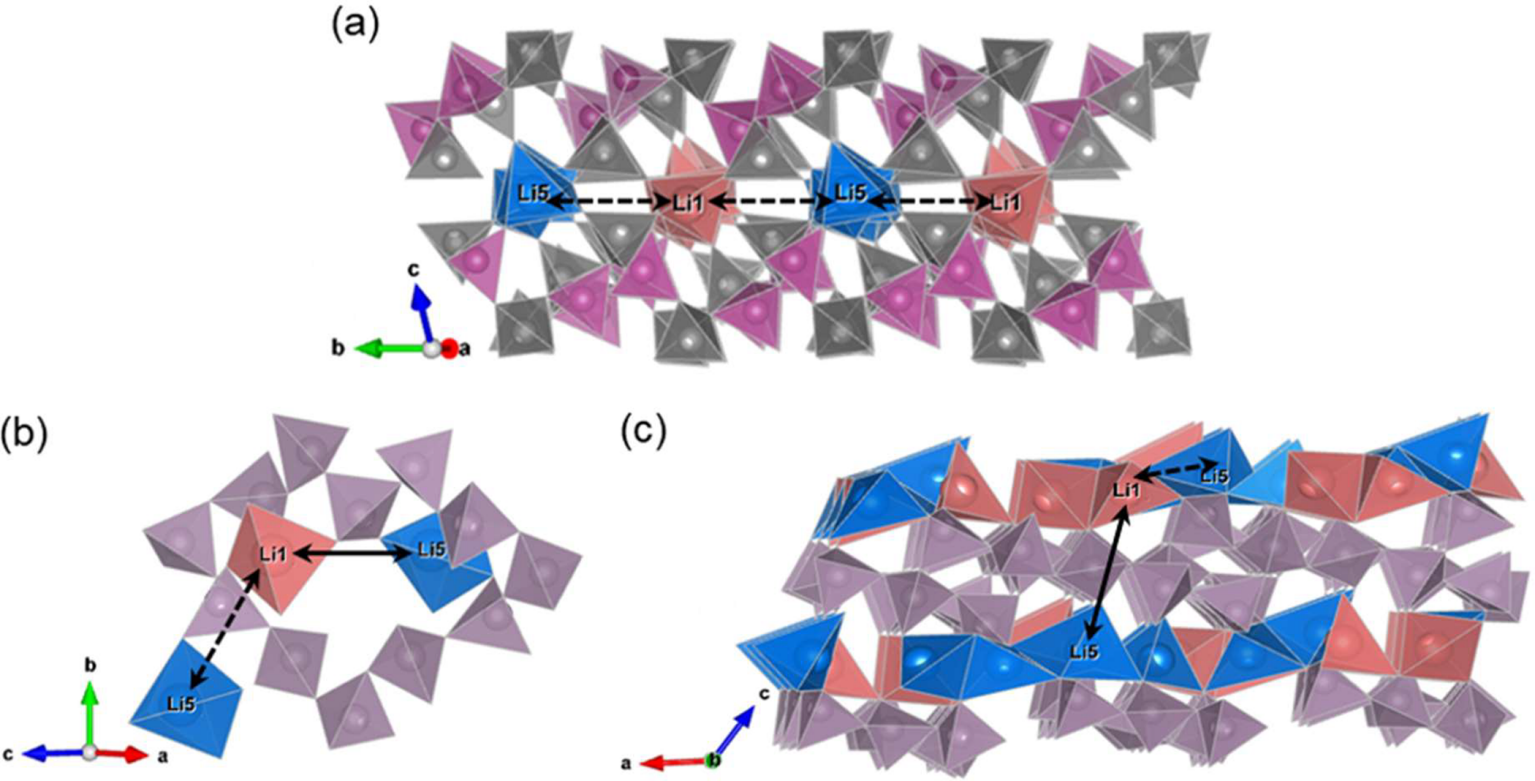
Visualization of the Li ion migration pathways in Li_3_P_5_O_14_ involving the most mobile sites (as seen from ^6^Li SLR measurements) Li1 and the five-coordinate Li5. (a) Intralayer Li ion pathway (dashed arrows) as shown in the *a-b* plane. (b) View of interlayer ion migration (full arrows) occurring between the 12-membered P_12_O_36_^12–^ rings, where only the Li atoms involved and one 12-membered ring are shown. An adjacent Li5 atom within the same layer as Li1, where migration occurs through the intralayer mechanism, is also shown for comparison. (c) View of the *a-c* plane in which both pathways can be visualized. A single Li3 atom that shares edges with Li1 has been omitted for the sake of clarity. The gray and purple tetrahedra represent internal and branching PO_4_^3–^ tetrahedra, respectively, while type A and type B Li polyhedra are colored red and blue, respectively. The possible Li migration pathway between sites Li5 and Li8 is equivalent to that between Li1 and Li5 shown in this figure.

While the ^7^Li SLR rate constants provide valuable information about the average Li ion mobility rates and dimensionality, ^6^Li SLR rates under MAS provide insights into the site specific Li ion motion. The ^6^Li SLR rates of the five different resonances remain largely constant below 338 K. However, at >338 K, the *T*_1_^–1^ values corresponding to the two peaks at −0.4 ppm (Li1) and −1.2 ppm (Li5 and Li8) begin to increase, which we tentatively attribute to increased Li ion mobility associated with these sites. Li1 occurs in the type A chains of Li_3_P_5_O_14_ edge sharing with adjacent LiO_4_ tetrahedra (Li3 and Li10). It was previously suggested through BVS mapping, arising from DFT data,^20^ that the ion mobility mechanism in Li_3_P_5_O_14_ occurred via two mechanisms, interlayer within the Li_6_O_16_^26–^ chains and also intralayer where local jumps occurred between the two types of Li_6_O_16_^26–^ chains. BVS mapping postulated that the local jumps between the two types of Li_6_O_16_^26–^ chains could occur through several possible pathways, one such pathway occurring via the Li5 distorted square pyramid sites. Therefore, exchange between Li1 and Li5 between Li_6_O_16_^26–^ chains is likely the driving force behind the increase in the ^6^Li SLR rates at >338 K. It should also be noted that the ^6^Li SLR rates for the two peaks at −0.4 ppm (Li1) and −1.2 ppm (Li5 and Li8) are noticeably greater across the entire temperature range. This observable cannot be explained by increased Li mobility, given the absence of temperature dependence of the SLR rates below 338 K. Therefore, the increased *T*_1_^–1^ values of the two peaks at −0.4 ppm (Li1) and −1.2 ppm (Li5 and Li8) are likely driven by another process. Given the fact that the environments for all Li sites in L_i3_P_5_O_14_ are tetrahedral LiO_4_ with very similar bond lengths and angles (with the exception of the five-coordinate Li5 site), we postulate this difference in *T*_1_^–1^ must be due to a change in environment during Li ion mobility. The interlayer pathway in Li_3_P_5_O_14_ occurs between 12-membered P_12_O_36_^12–^ rings that provide a window that mobile Li ions can traverse, and the environment inside of the P_12_O_36_^12–^ rings would be extremely different from that in which the Li ions usually reside, likely giving rise to a change in SLR rate. The described inter- and intralayer pathways that occur via exchange between Li1 and Li5 can also be accessed via exchange between Li5 and Li8; however, because this pathway cannot be probed directly due to the lack of resolution in the corresponding ^6^Li NMR resonances, the discussion is focused on the pathway between Li1 and Li5. Because Li1 and Li8 reside in the A type Li_6_O_16_^26–^ chain while Li5 resides at an adjacent position in the B type chain, in the *a-b* plane and along the *c*-axis, it is quite likely that these sites play a pivotal role in both the 2D intralayer (Figure 8a) and the resulting 3D interlayer ion mobility (Figure 8b,c).

### ^7^Li Spin-Alignment Echo NMR

*T*_1_ and *T*_1ρ_ measurements probe Li ion mobility on megahertz and kilohertz scales, respectively, and slower motion on the hertz and subhertz scale can be obtained from SAE NMR, an approach that also has the added benefit of allowing for direct measurement of τ_c_^–1^ at any given temperature.^70−73^ The underlying principle of SAE NMR spectroscopy is similar to that of 2D EXSY NMR,^74^ where instead of utilizing a change in the chemical shift interactions, SAE NMR takes advantage of a change in the interactions between the quadrupole moment of the nucleus with the electric field gradient tensor when exchange occurs. ^7^Li SAE NMR spectra of Li_3_P_5_O_14_ were recorded as a function of τ_m_ at temperatures of 295, 330, and 373 K, and the resulting data capturing the echo amplitude decays are shown in Figure 8. If sufficiently long τ_m_ values are sampled, the resulting echo amplitudes have a two-step decay, where the first decay step is directly characterized by the Li ion jump processes between electronically inequivalent sites and the second decay step is characterized by the quadrupolar component of the SLR, *T*_1,Q_. Thus, only the part of the curve governed by Li ion motion is captured in the decay curves of Li_3_P_5_O_14_, because the first decay step is observed, while longer τ_m_ values would be required for the second decay step. The solid lines in Figure 8 show fits to eq 3 through which Li ion τ_c_^–1^ values of 0.6(1), 7(1), and 114(8) s^–1^ were obtained at 295, 330, and 373 K, respectively. A linear slope among these three data points yields an activation barrier of 0.62(5) eV, in good agreement with the values obtained from the ^7^Li line narrowing and SLR measurements.

### NMR-Derived Li^+^ Ion τ^–1^ Values

**Figure 9:**
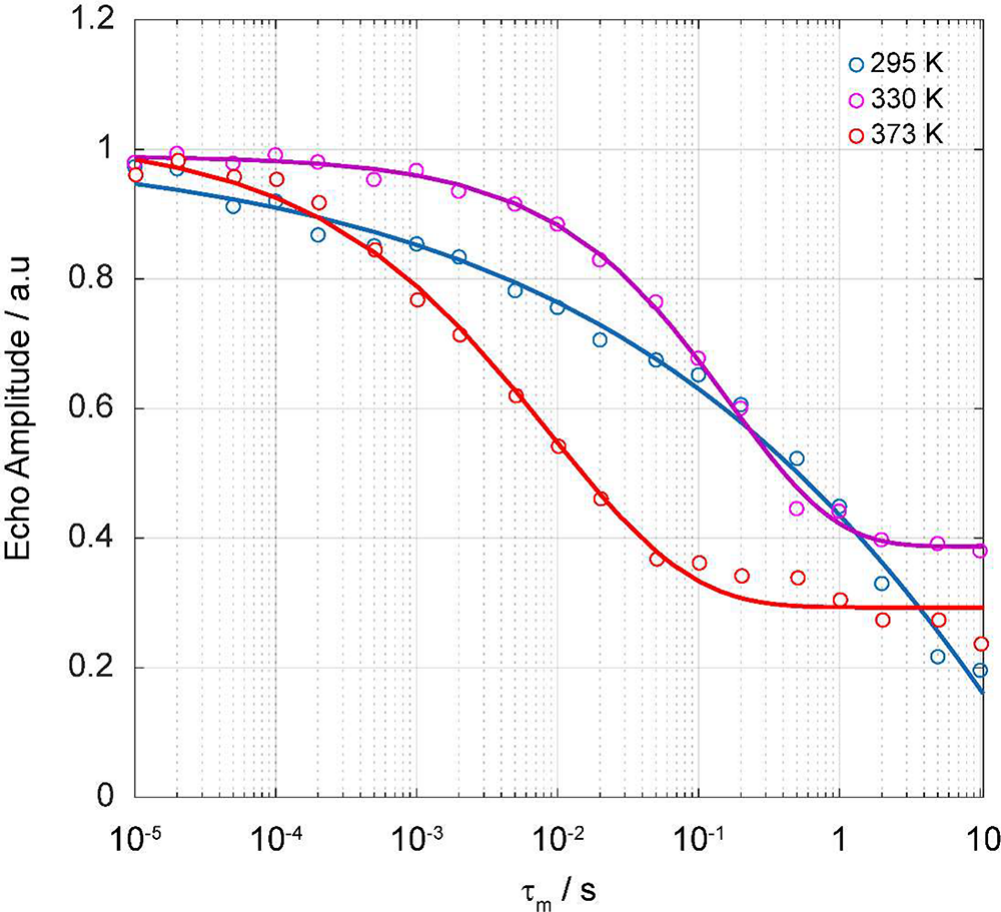
^7^Li SAE NMR echo amplitude as a function of τ_m_ at 295, 330, and 373 K. Solid lines show fits to the one-time correlation function (eq 3) with stretch exponential values (γ) of 0.62, 0.56, and 0.65 for the three temperatures, respectively.

**Figure 10:**
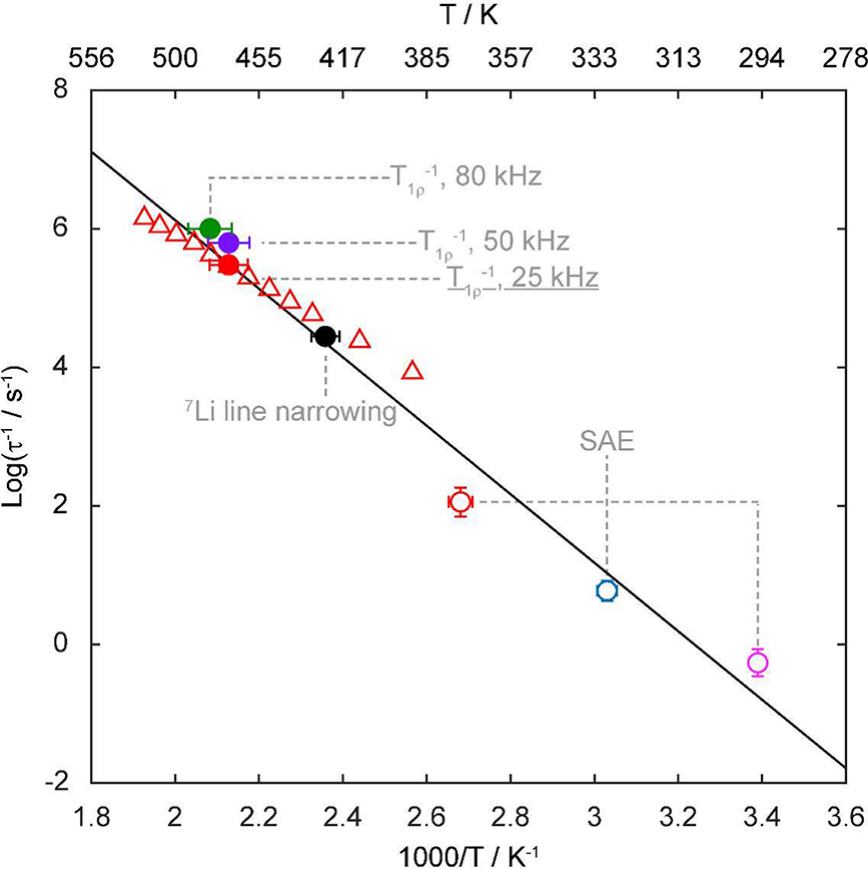
Arrhenius plot of Li jump rates τ^1^ showing data points obtained from BPP simulations (red triangles; ω_1_/2π = 25 kHz), extracted from the onset of ^7^Li line narrowing of the variable-temperature ^7^Li NMR spectra (black circle, previously reported data),^20^^7^Li SAE experiments (empty colored circles, Figure 9), and SLR rates in the rotating frame (*T*_1ρ_^–1^) experiments (filled colored circles, Figure 7) at spin-lock frequencies ω_1_/2π of 25 kHz (red), 50 kHz (purple), and 80 kHz (green), respectively. The label for the spin-lock frequency used in the BPP simulation for this figure is underlined. Errors in jump rate τ^–1^ are within the data points.

NMR-derived jump rates τ^–1^ obtained from the previously recorded ^7^Li line narrowing experiments, SAE (Figure 9), relaxometry experiments (Figure 7), and BPP simulation for an ω_1_/2π of 25 kHz are plotted against reciprocal temperature in Figure 10 (data for ω_1_/2π values of 50 and 80 kHz are given in Figures S13 and S14, respectively). There is an excellent agreement between the τ^–1^ values obtained from ^7^Li line narrowing spectra, SAE, and relaxometry data, and these data agree reasonably well with the τ^–1^ values obtained from the BPP simulations. An activation barrier for Li ion mobility in Li_3_P_5_O_14_ of 0.9(2) eV is obtained from the slope of the experimentally obtained data points (excluding the BPP simulations, which appear to overestimate τ_c_^–1^ below ∼460 K), noting that particularly large degrees of uncertainty are observed here, likely due to the combination of various methods used. Hence, the energy barriers obtained from ^7^Li line narrowing and SAE and SLR experiments are likely more informative. The activation barriers obtained via the various spectroscopic methods used here are summarized in Table 3 and are consistently approximately around 0.6-0.7 eV. In particular, there is a strong agreement between *E*_a_ values obtained through ^7^Li line narrowing, *T*_1ρ_^–1^ on the high-temperature flank, BPP simulation, and SAE experiments. Values obtained for ^7^Li *T*_1ρ_^–1^ on the low-temperature flank and through combining all jump rate values obtained through the various methods, however, are not in full agreement with the range of 0.6-0.7 eV. The discrepancy in *T*_1ρ_^–1^ on the low-temperature flank is likely a result of the large errors associated with collecting these data due to the very long *T*_1ρ_ times that require continuous rf pulsing for a duration that exceeds the probe capabilities and are hence not measurable. The discrepancy in the activation energy obtained from the jump rate plots (Figure 10 and Figures S13 and S14) is likely due to the combination of various different methods that probe dynamics in largely different ways. The activation energy obtained from alternating current impedance spectroscopy (ACIS) [0.42(8) and 0.43(7) eV for the bulk and total conductivity, respectively] is cautiously comparable to the average value obtained from NMR (∼0.6 eV) given the largely different methods used and differing length scales probed (bulk in ACIS vs local ion hops in NMR), experimental uncertainty, and the complex ion pathways in Li_3_P_5_O_14_.

**Table 3:** Summary of the Activation Barrier for Li_3_P_5_O_14_ Extracted from the Bulk Conductivity of the ACIS,^20^^7^Li Motional Narrowing,^75^ SAE NMR from Figure 9, and SLR Data in the Laboratory Frame (*T*_1_) and Rotating Frame (*T*_1ρ_) from Figure 7^a^

activation energy (eV)							
ACIS^20^	Waugh-Fedin^20^	*T*_1_	*T*_1ρ,LT_	*T*_1ρ,HT_	BPP fit	SAE	jump rate
0.42	∼0.6	0.58(7)	0.9(2)	0.7(1)	0.60(6)	0.62(5)	0.9(2)

a Activation barriers on the high- and low-temperature flanks of the BPP curve are quoted, along with the activation barrier obtained from the jump rate plots shown in Figure 10 and Figures S13 and S14.

## Conclusion

Li_3_P_5_O_14_ represents a new type of fast lithium conducting oxide-based solid electrolyte candidate. This phase possesses an ultraphosphate chemical structure whose local Li and P environments have been probed through ^6,7^Li and ^31^P MAS NMR. We also employed a range of complementary NMR approaches to quantify the Li ion dynamics and identify the Li ion mobility pathway in Li_3_P_5_O_14_. This work illustrates the importance of NMR in the characterization of the structure of high-performance solid electrolytes, as well as the Li ion mobility pathways. The latter allows for the identification of beneficial structural features for long-range Li ion motion and hence the further development of solid electrolyte candidates.

The local Li and P environments were investigated via ^6^Li and ^31^P MAS NMR in conjunction with DFT calculations to assign a large number of distinct sites. It was shown that the eight ^31^P NMR resonances with the lowest chemical shift also possessed the smallest CSA, leading to the assignment of these resonances to phosphate groups that possess a high degree of connectivity. ^31^P-^31^P INADEQUATE NMR spectra allowed for the complete assignment of the ultraphosphate layer in Li_3_P_5_O_14_, where the PO_4_^3–^ tetrahedra remain relatively immobile with little to no phosphate reorientation, as shown from static variable-temperature (VT) ^31^P NMR as well as ^31^P SLR measurements.

A number of ^7^Li NMR approaches were employed to capture the Li ion dynamics in Li_3_P_5_O_14_. Static ^7^Li VT NMR, SAE NMR, and relaxometry enabled the quantification of the Li ion dynamics. Moreover, the frequency dependence of the ^7^Li SLR rates in the rotating frame of reference allowed for the identification of a 3D Li ion pathway in Li_3_P_5_O_14_ that occurs along the Li_6_O_16_^26–^ chains as well migrating between chains. ^6^Li SLR and ^6^Li-^6^Li EXSY measurements permit the identification of the more mobile sites Li1 and Li5 that likely migrate between Li_6_O_16_^26–^ chains, in the *a-b* plane and along the *c*-axis. While this pathway is also possible for exchange between Li5 and Li8, we cannot experimentally probe this exchange due to the lack of resolution between these sites. However, the possibility of two separate inter- and intralayer Li ion migration pathways that occur via exchange with the only five-coordinate Li site implies that this site with a greater coordination number facilitates inter- and intralayer migration in Li_3_P_5_O_14_.

## Data Availability

The research data supporting this publication, including data from ^31^P MAS, ^31^P-^31^P refocused INADEQUATE, ^6^Li MAS, and static ^6^Li, ^7^Li, and ^31^P VT experiments and jump rate plots, can be accessed from the University of Liverpool Data catalogue available at https://datacat.liverpool.ac.uk/2714/.

## Supporting Information Available

Crystal structures of different arrangements of phosphates, a plot comparing CASTEP-calculated ^31^P isotropic chemical shieldings and experimentally observed ^31^P isotropic chemical shifts, ^31^P MAS NMR spectra with varying recycle delays, ^31^P MAS NMR spectra and deconvolutions showing the full spectral width, crystallographic P labels vs ^31^P assignments, ^31^P-^31^P INADEQUATE NMR spectra centered at the first set of spinning side bands, 1D traces of INADEQUATE spectrum, overview of ^6^Li calculated and experimental NMR parameters, ^6^Li MAS NMR deconvolutions with varying numbers of resonances, temperature dependence of the ^6^Li shifts, ^6^Li-^6^Li EXSY NMR spectra, magnified view of the ^7^Li relaxometry data fits, static ^31^P NMR spectra, ^7^Li relaxometry BPP curves, and ^7^Li jump rate plots displaying calculated values at ω_1_/2π values of 50 and 80 kHz (PDF)
