# Supplementary material for: Revealing the Local Structure and Dynamics of the Solid Li Ion Conductor Li3P5O14
Source: Chem Mater. 2024 Jul 29;36(16):7703–18. doi: 10.1021/acs.chemmater.4c00727 (PMC11360135; doi:10.1021/acs.chemmater.4c00727)
Supplement: Supplementary file 1 — cm4c00727_si_001.pdf [file cm4c00727_si_001.pdf]

Supporting Information For

Revealing the Local Structure and Dynamics of the Solid Li-Ion Conductor $Li_3P_5O_{14}$

Benjamin B. Duff,[a,b] Lucia Corti,[a] Bethan Turner,[a] Guopeng Han,[a] Luke M. Daniels,[a] Matthew J. Rosseinsky,[a,c] and Frédéric Blanc[a,b,c*]

[a] Department of Chemistry, University of Liverpool, L69 7ZD Liverpool, UK. [b] Stephenson Institute for Renewable Energy, University of Liverpool, L69 7ZF Liverpool, UK. [c] Leverhulme Research Centre for Functional Materials Design, Materials Innovation Factory, University of Liverpool, L7 3NY Liverpool, United Kingdom

*Corresponding Author: frederic.blanc@liverpool.ac.uk

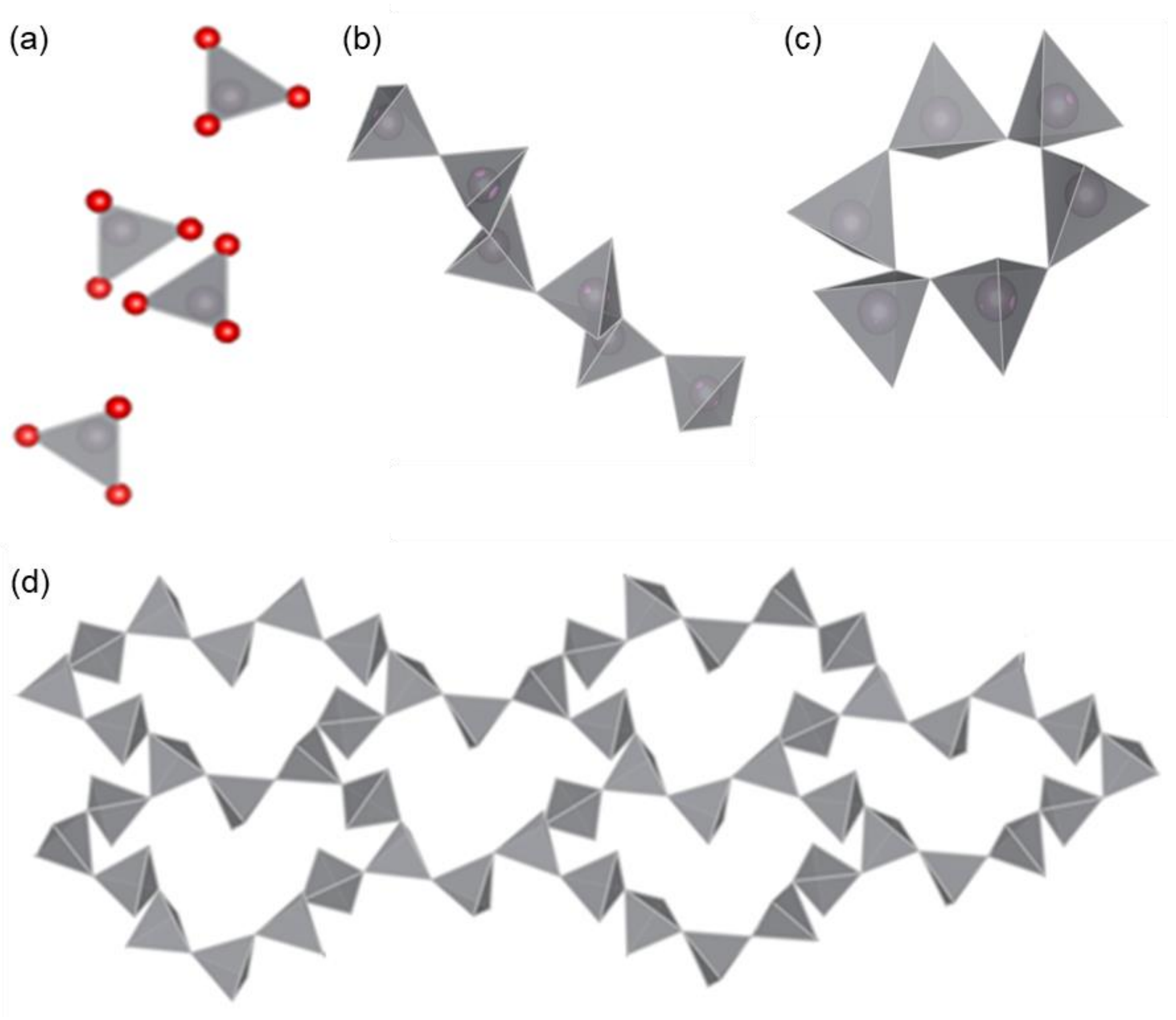

Figure S1 Arrangement of PO_4^{3-} tetrahedra in the four types of phosphates. (a) isolated PO_4^{3-} tetrahedra in orthophosphate Li_3PO_4. (b) 1D 2-conneted PO_3^- chain in polyphosphate $LiPO_3$. (c) 2D connected single $P_6O_{18}^{6-}$ ring in the cyclophosphate $Al_2P_6O_{18}$ and (d) $P_{20}O_{56}^{12-}$ chains in $Li_3P_5O_{14}$ form an ultraphosphate layer. O atoms in (a) only are shown in red and PO_4^{3-} tetrahedra are shown in grey. Structural parameters are taken from references [1,2] and [3] for panels (a-c), respectively, and replotted in VESTA.

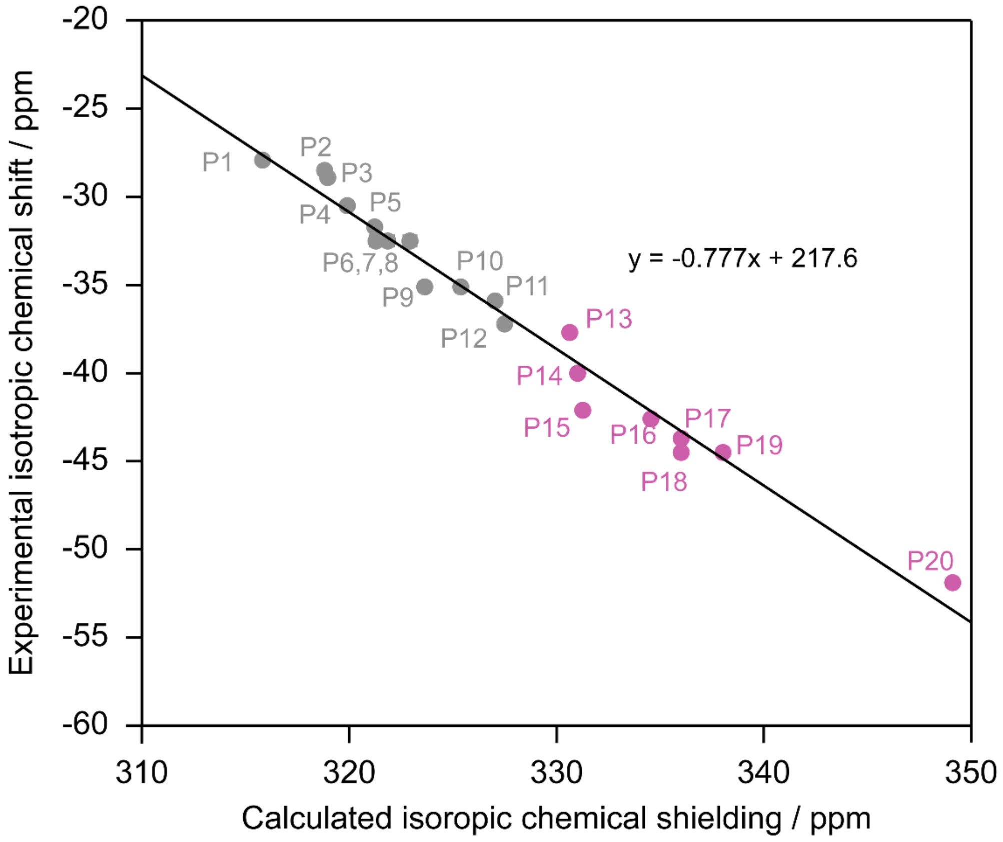

Figure S2 Comparison between CASTEP calculated ^{31}P isotropic chemical shieldings and the experimentally observed ^{31}P isotropic chemical shift from the MAS NMR spectrum of $Li_3P_5O_{14}$ (**Figure 2**) where the points are colour coded corresponding to PO_4^{3-} groups that bridge to two and three other PO_4^{3-} tetrahedra are in grey and pink respectively. The solid black line corresponds to a linear fit of the data and the majority of error bars are within the data point.

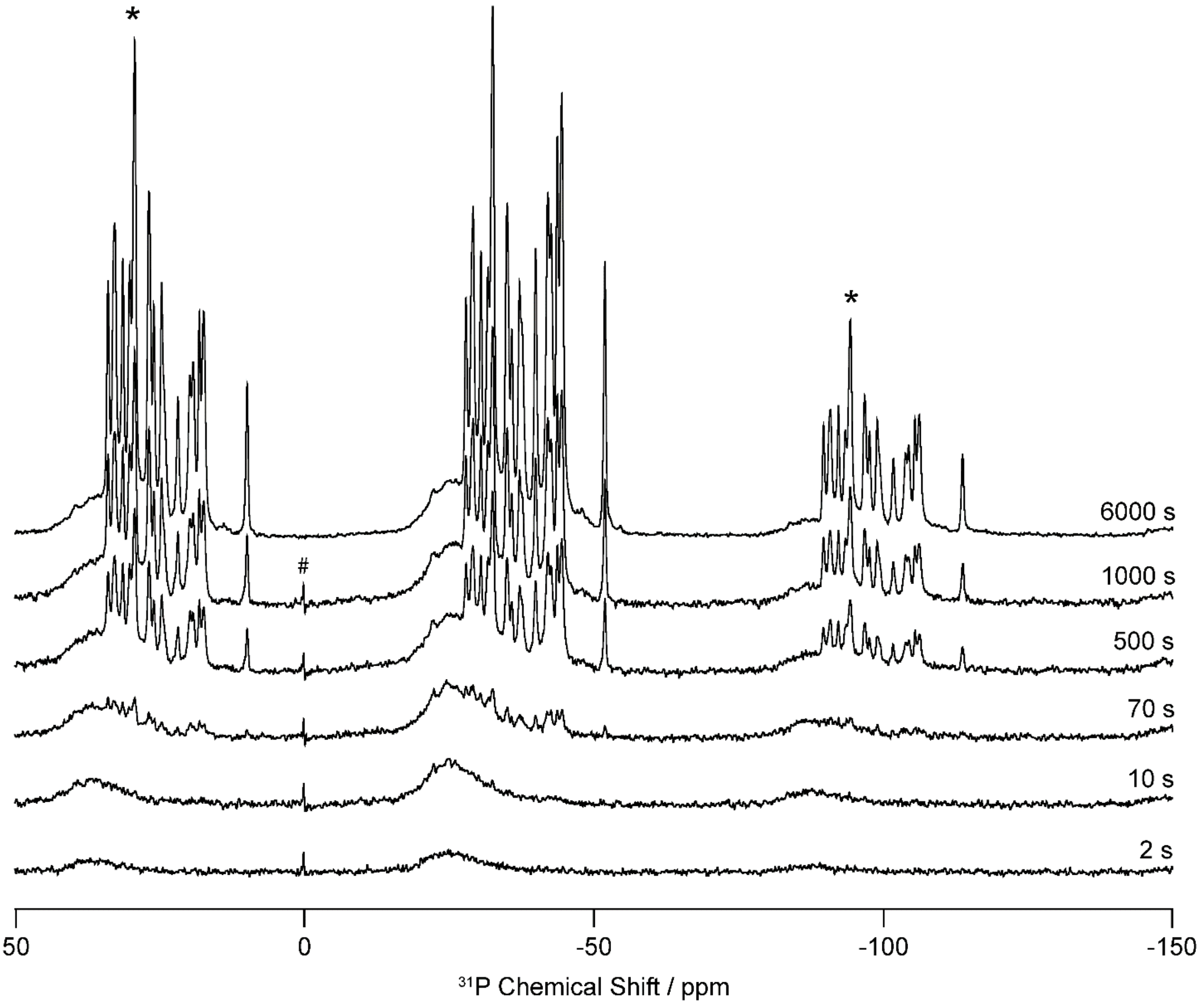

Figure S3 ^{31}P magic angle spinning (MAS) NMR spectra of the $Li_3P_5O_{14}$ phase as a function of the recycle delay, spectra shown are obtained from the individual spectra from the saturation recovery experiment. A small fraction (~ 12 % from integral) of amorphous phase is observed at short recycle delays. Spectra recorded with recycle delays of 2, 10, 70, 500, 1000 and 6000 s are shown. Asterisk symbols (*) and the hash symbol (#) denote spinning sidebands and the carrier frequency respectively.

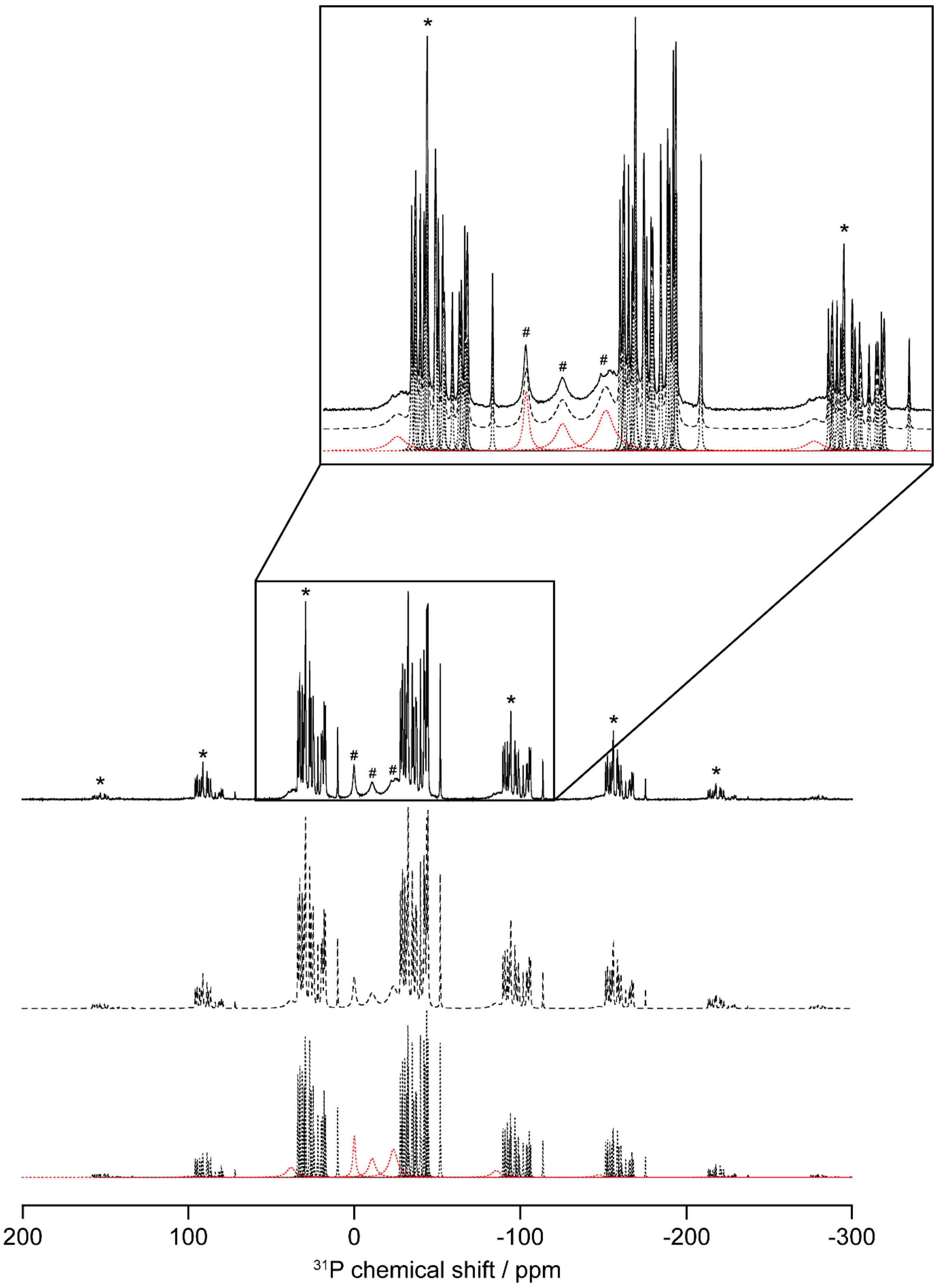

Figure S4 ^{31}P MAS spectrum of $Li_3P_5O_{14}$ showing the first set of spinning sidebands. The experimental spectrum (full black line), total fit (dashed black line), and spectral deconvolution (dotted lines) are shown. Hash symbols (#) denote impurities, with the peaks between ~ 0 and -15 ppm (ref line) corresponding to impurities arising from sample degradation due to exposure to atmosphere.

Table S1 Comparison between the labelling of the crystallographic P sites obtained from ^{31}P NMR with the previously reported diffraction data (ICSD 114286)[4]

^{31}P NMR assignments	Crystallographic P sites
1	14
2	1
3	3
4	13
5	9
6,7,8	11,17,18
9,10	7,8
11	20
12	4
13	12
14	15
15	5
16	16
17	19
18,19	6,10
20	2

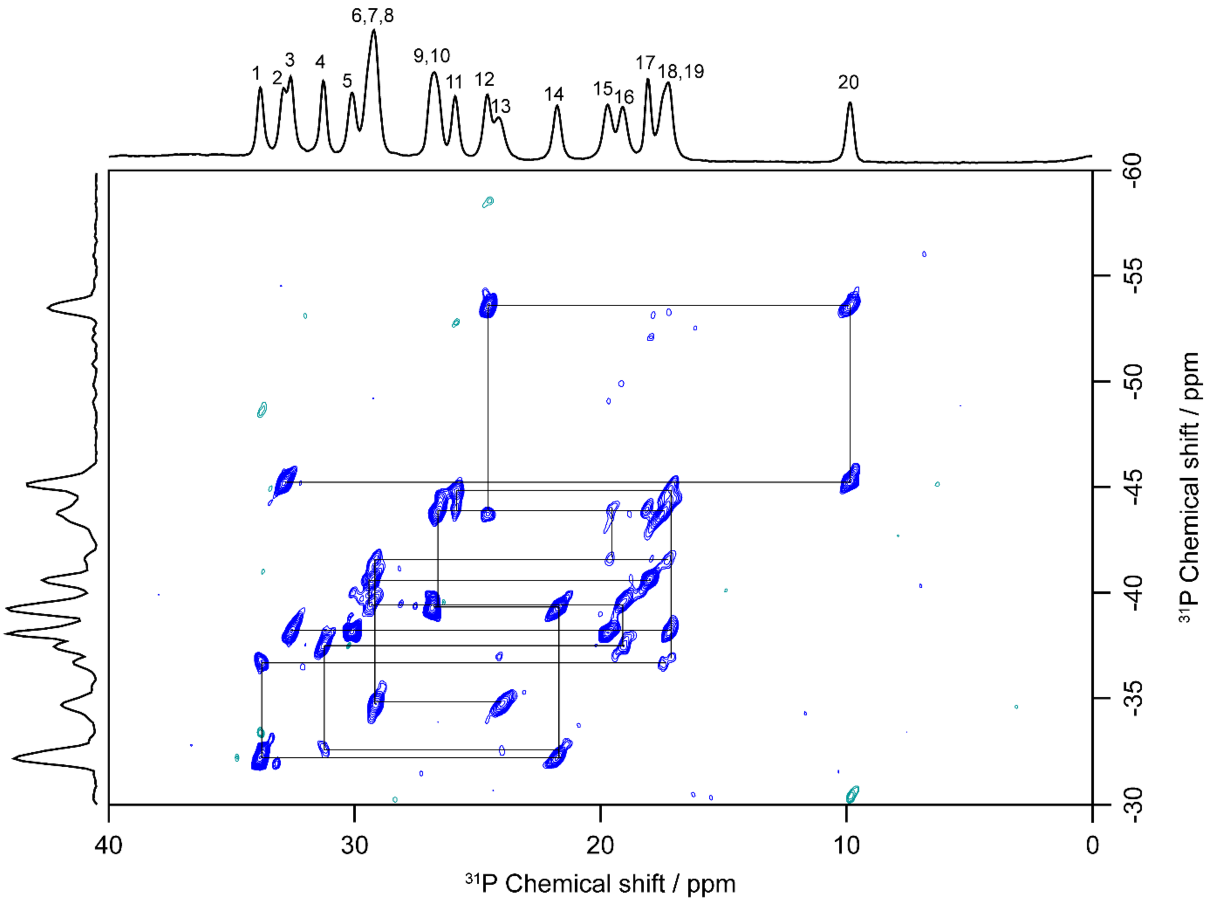

Figure S5 2D refocussed INADEQUATE ^{31}P-^{31}P NMR spectrum of $Li_3P_5O_{14}$ showing the observable correlations with black lines collected with echo durations of 6 ms. The

spectral window focusses on the region of the first spinning sideband at higher chemical shift than the isotropic region shown in the main text (**Figure 4(b)**).

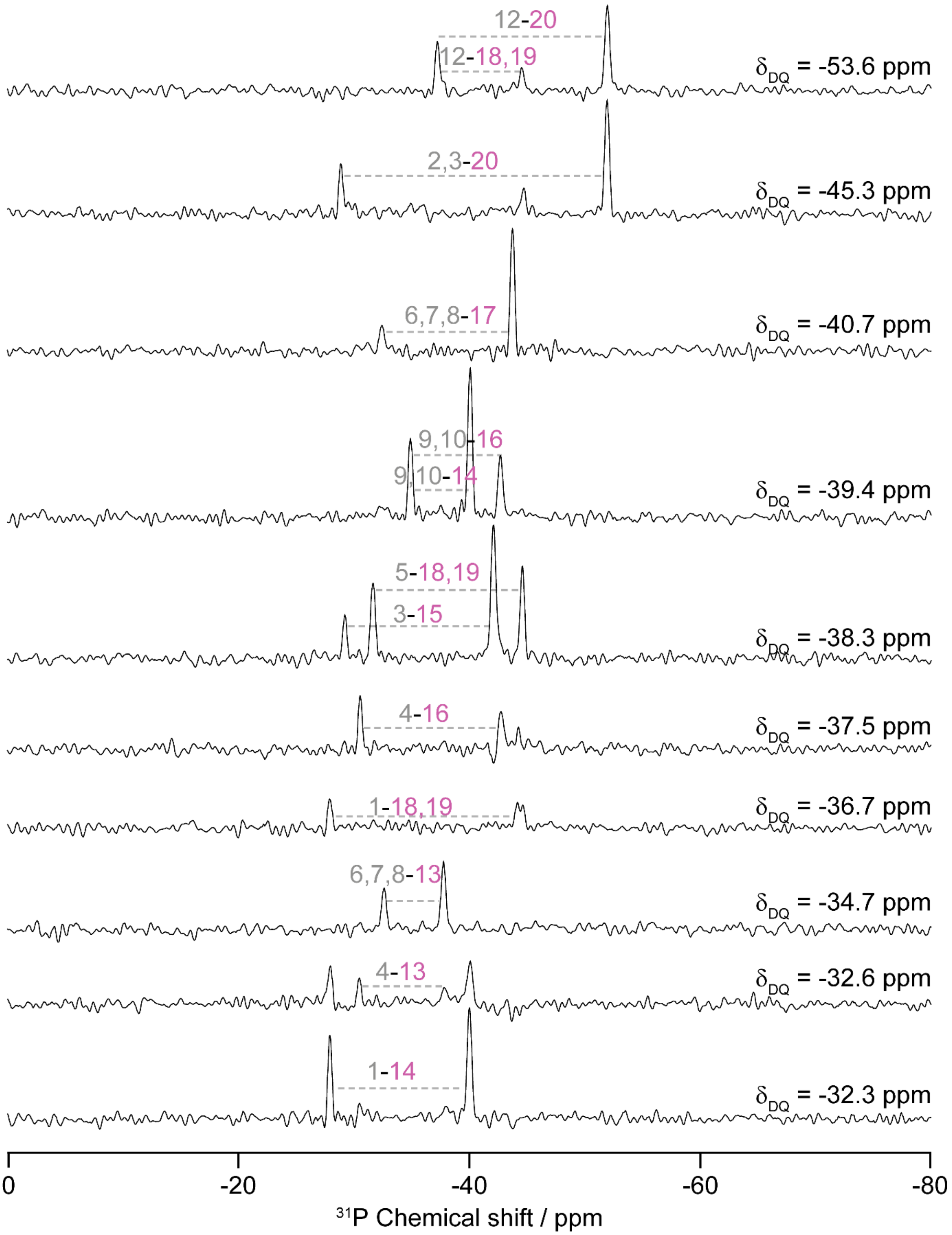

Figure S6 Selected horizontal traces of the ^{31}P-^{31}P refocussed INADEQUATE NMR spectrum of $Li_3P_5O_{14}$ (**Figure 4(b)**) showing the assignments of the correlations where PO_4^{3-} groups that bridge to two and three other PO_4^{3-} tetrahedra are coloured in grey

and pink respectively. The corresponding double quantum frequency of each slice is given in the figure.

Table S2 Summary of the computed and observed ^{6}Li NMR observables for $Li_3P_5O_{14}$, where computed parameters are shown in plain text, while experimentally observed parameters are shown in bold text. Computed parameters were obtained using the GIPAW method implemented in CASTEP.

Assignment	Calculated shielding	$\delta_{iso,cs}$ / ppm	$\delta_{aniso,cs}$ / ppm	η
1	89.8	3.3/**-0.4**	-1.0	0.90
2	90.8	2.4/**-1.4**	-6.2	0.88
3	90.2	2.9/**-1.0**	7.3	0.51
4	89.9	3.2/**-0.7**	-3.4	0.19
5	90.4	2.7/**-1.2**	-5.5	0.55
6	90.3	2.8/**-1.0**	3.6	0.81
7	89.8	3.3/**-0.7**	4.3	0.39
8	90.5	2.6/**-1.2**	5.1	0.50
9	90.6	2.5/**-1.4**	5.1	0.32
10	90.4	2.8/**-1.0**	4.9	0.54
11	90.3	2.9/**-1.0**	-6.8	0.81
12	90.2	2.9/**-1.0**	5.0	0.46

[a] Experimental and computed isotropic shifts were obtained via the deconvolution of the ^{6}Li NMR spectrum in **Figure 5** and from the computed σ_{iso} values via the expression $\delta_{iso,cs} = \sigma_{ref} + m\sigma_{iso}$ respectively.
[b] Computed $\delta_{aniso,cs}$ values were obtained via the calculated $\sigma_{aniso,cs}$ Haeberlen convention, such that $\sigma_{aniso,cs} = \sigma_{zz} - 1/2(\sigma_{xx} + \sigma_{yy})$ and $\delta_{aniso,cs} = m\sigma_{aniso,cs}$.
[c] Computed η values were obtained via the Haeberlen convention, such that $\eta = (\sigma_{yy} - \sigma_{xx})/(\sigma_{zz} - \sigma_{iso})$.

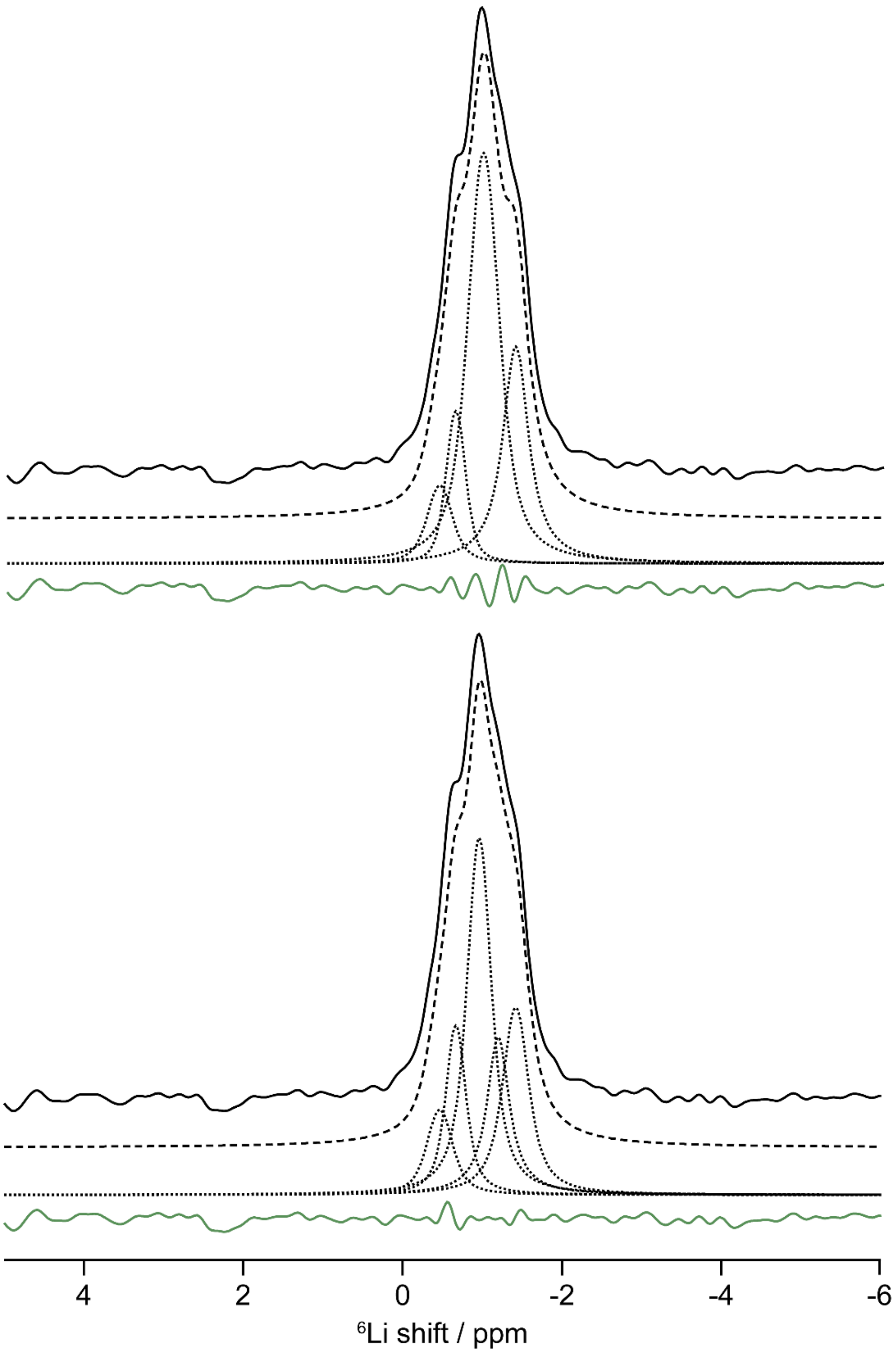

Figure S7 ^{6}Li MAS NMR spectra of $Li_3P_5O_{14}$ comparing a four-fit model (top) to the five-fit model (bottom) used throughout this work. The experimental spectra (full black lines), total fit (dashed lines), spectral deconvolution (dotted lines) and residual fits (full green lines) are shown.

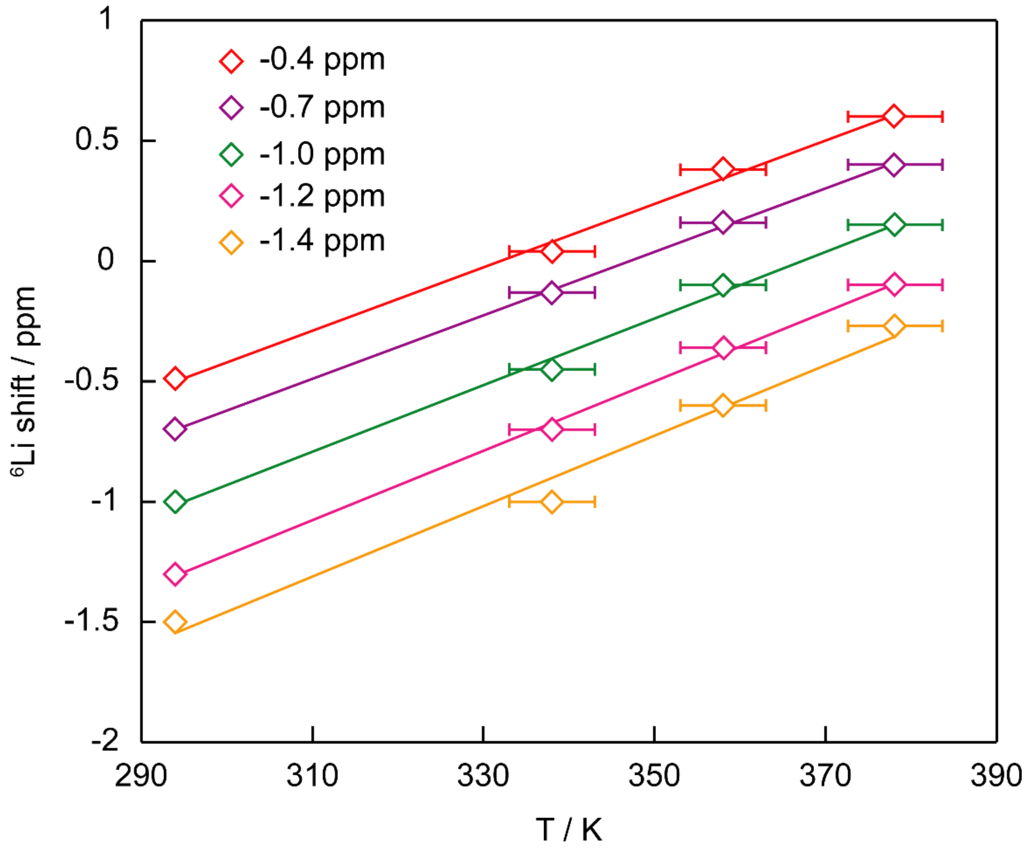

Figure S8 Temperature dependence of the different deconvoluted ^{6}Li shifts collected at 20 T and $\omega_r/2\pi$ = 10 kHz. Data points are colour coded the same as in **Figure 7**. Errors in the temperature are obtained via the same method as described in the experimental section. Errors in the shift are omitted for clarity but can be assumed to be roughly half of the peak width in **Figure 6** (0.1-0.2 ppm on average).

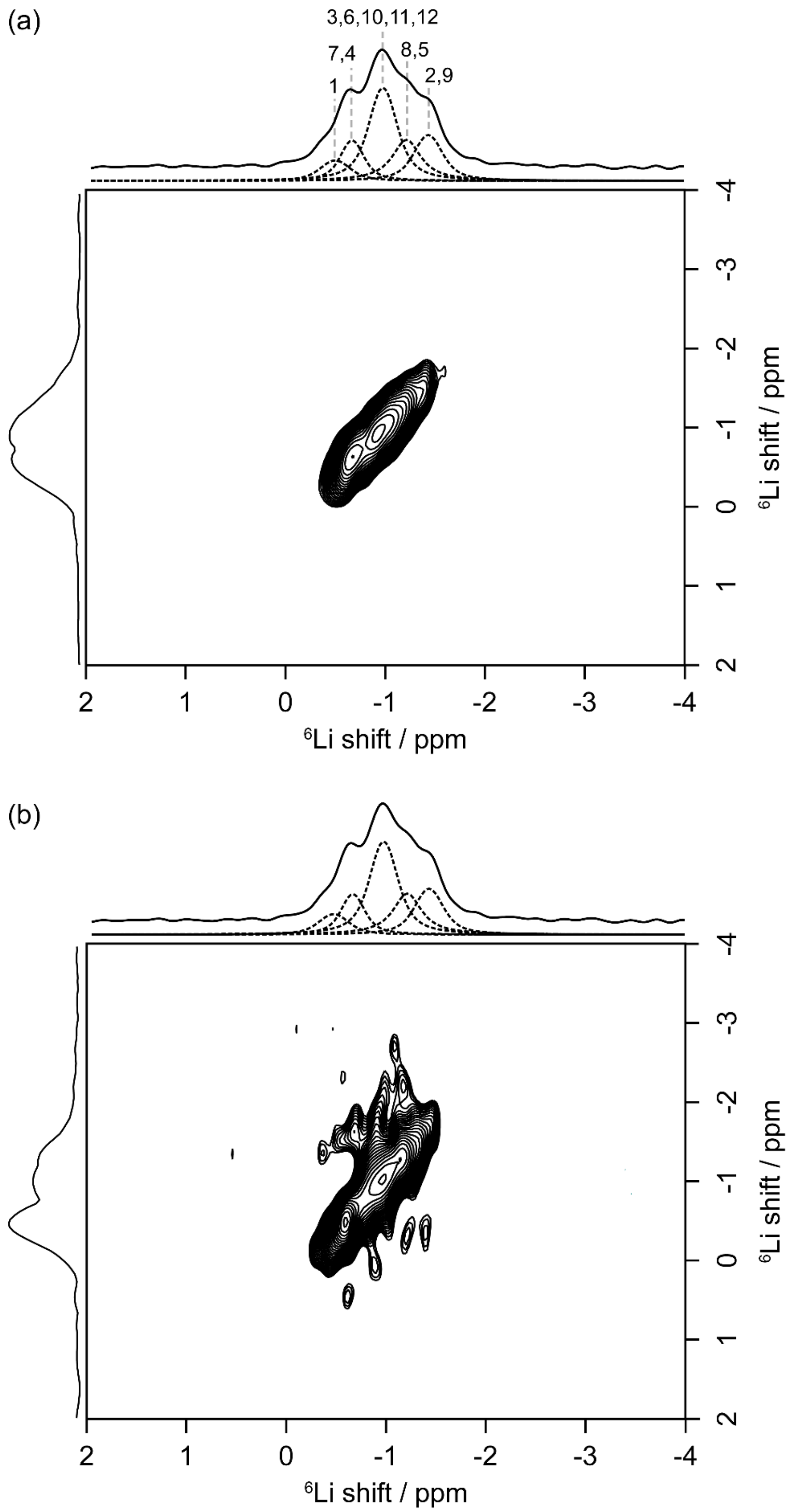

Figure S9 2D ^6Li-^6Li Exchange spectroscopy NMR spectra of $Li_3P_5O_{14}$ at mixing times of (a) 0 and (b) 5 s where the spectrum in the horizontal dimension displays the 1D experimental spectrum with the spectral deconvolution (dotted lines) and spectral assignment.

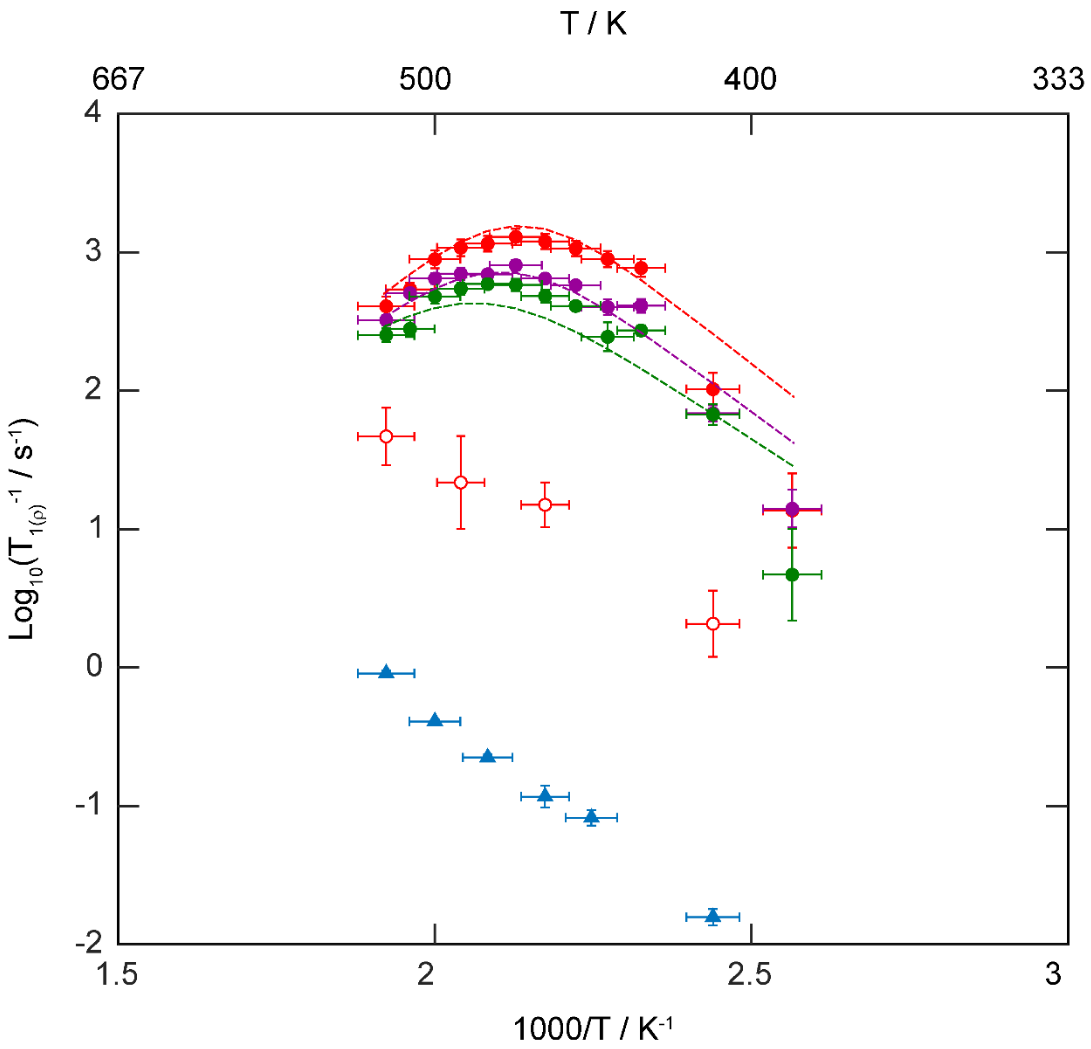

Figure S10 Magnified view of the Arrhenius plots focussing on the ^{7}Li NMR SLR rate constants in the rotating frame ($T_{1\rho}^{-1}$) at $\omega_1/2\pi$ = 25 (red circles), 50 (purple circles) and 80 kHz (green circles). Coloured dashed lines outline the fitting of the experimental data to **equation 6**.

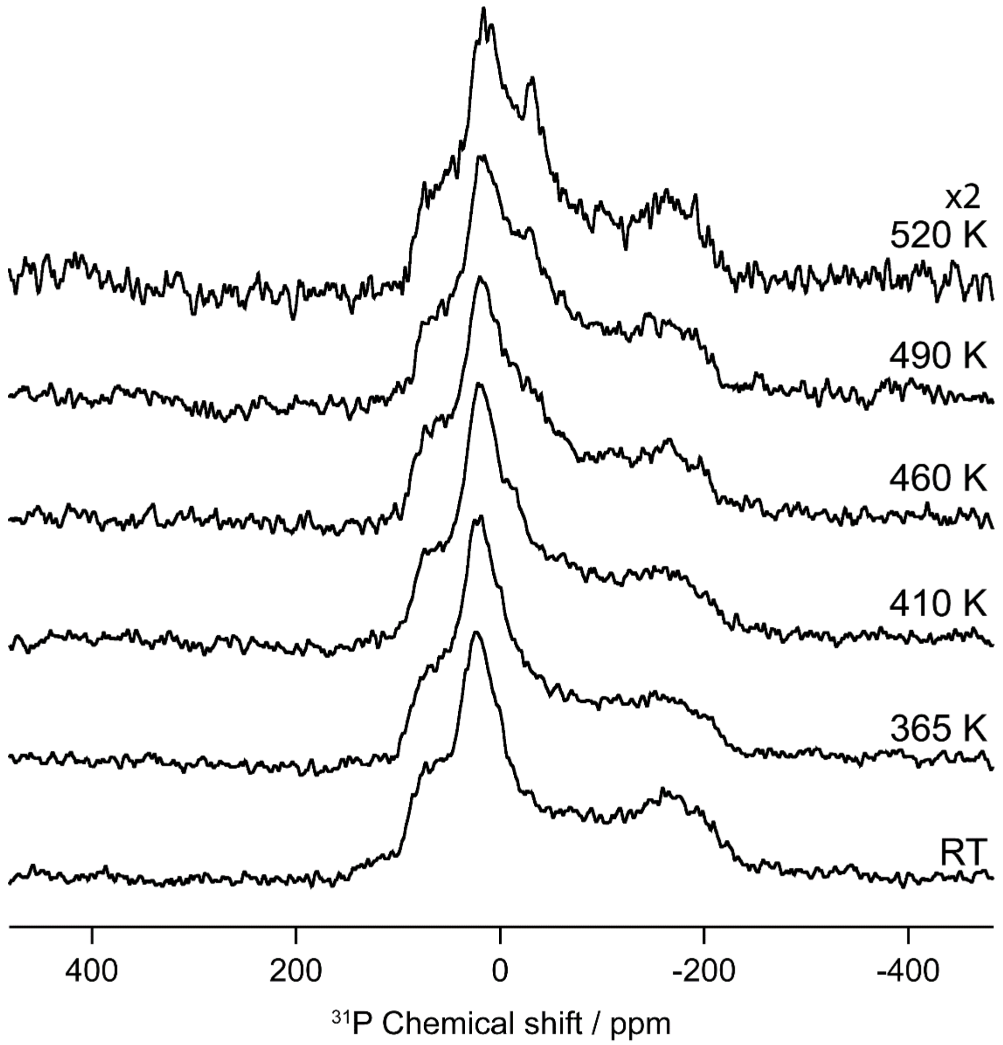

Figure S11 Static ^{31}P NMR spectra of $Li_3P_5O_{14}$ as a function of temperature. The CSA patterns remain largely unchanged with increasing temperature, indicating a lack of PO_4^{3-} reorientation motion. The spectra at 520 K is magnified by a factor of two to factor in the lower number of scans used.

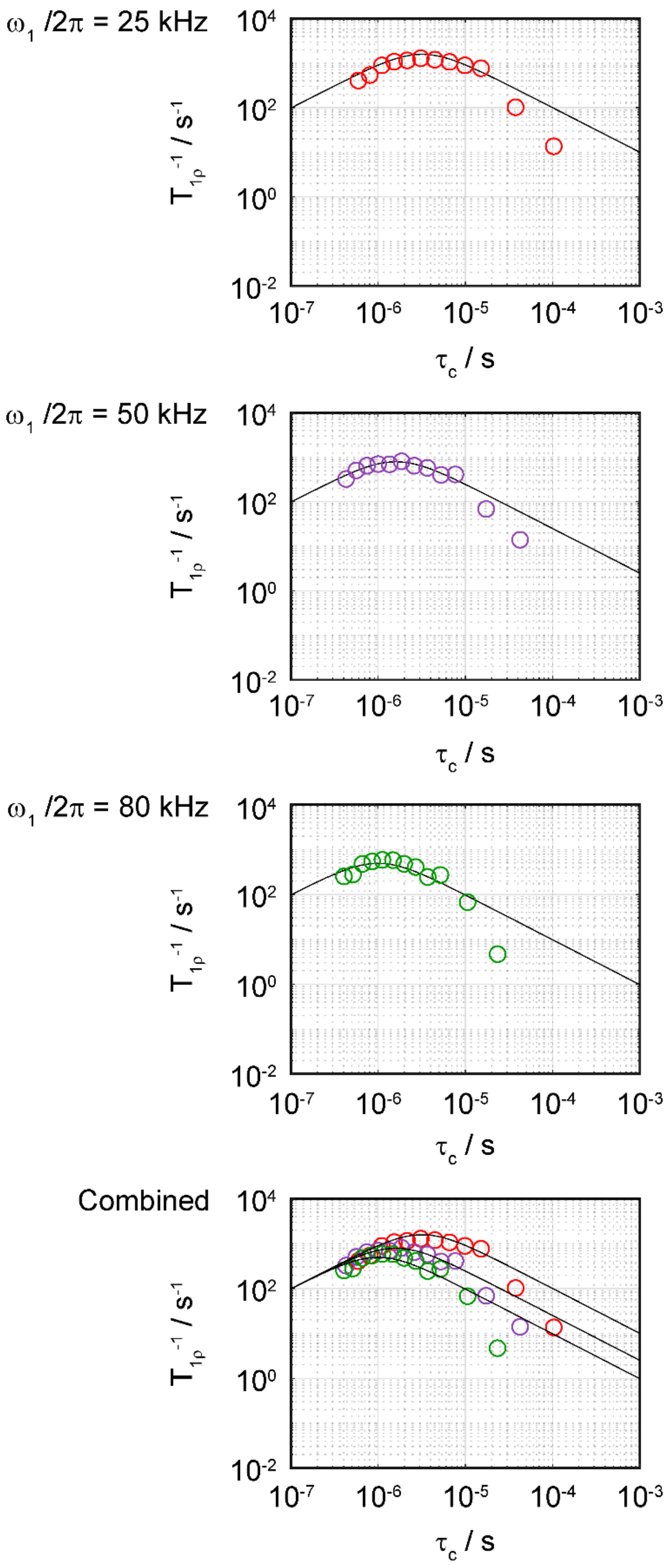

Figure S12 ^{7}Li $T_{1\rho}^{-1}$ versus τ_c for $Li_3P_5O_{14}$, collected at spin-lock frequencies of $\omega_1/2\pi$ of 25 (red), 50 (purple) and 80 kHz (green), the solid lines are those obtained from **equation 6** using the experimentally determined local field fluctuation term of 7 x 10^8 Hz^2 (average value from **Table 2**).

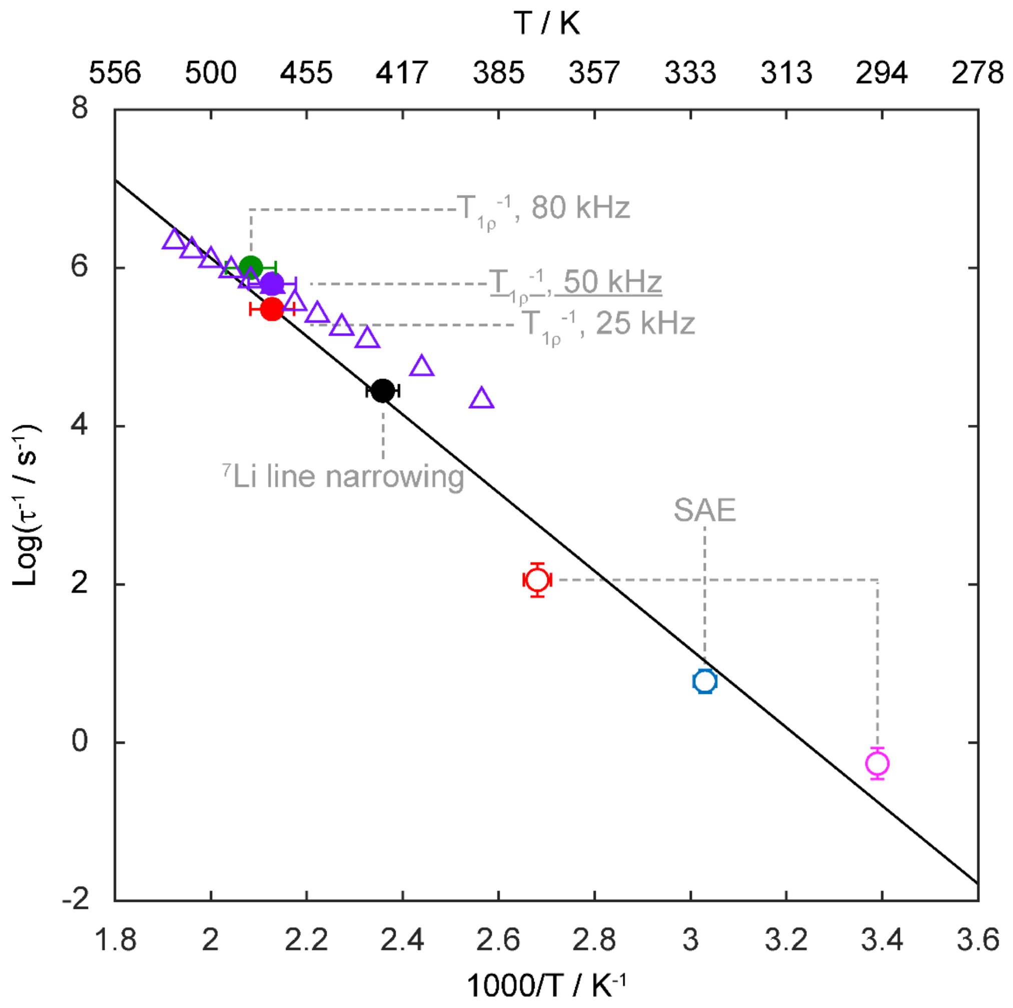

Figure S13 Arrhenius plot of Li jump rates τ_c^{-1} showing the points obtained from BPP simulations (purple triangles, $\omega_1/2\pi = 50$ kHz) Data were extracted from the onset of ^{7}Li line narrowing of the variable temperature ^{7}Li NMR spectra (black circle, previously reported data[4]), ^{7}Li SAE experiments (empty coloured circles, **Figure 9**), SLR rates in the rotating frame ($T_{1\rho}^{-1}$) experiments (filled coloured circles, **Figure 7**) at spin lock frequencies $\omega_1/2\pi$ of 25 (red), 50 (purple) and 80 kHz (green), respectively. The label for the spin locking frequency used in the BPP simulation for this figure is underlined. Errors in the jump rate τ_c^{-1} are within the data points.

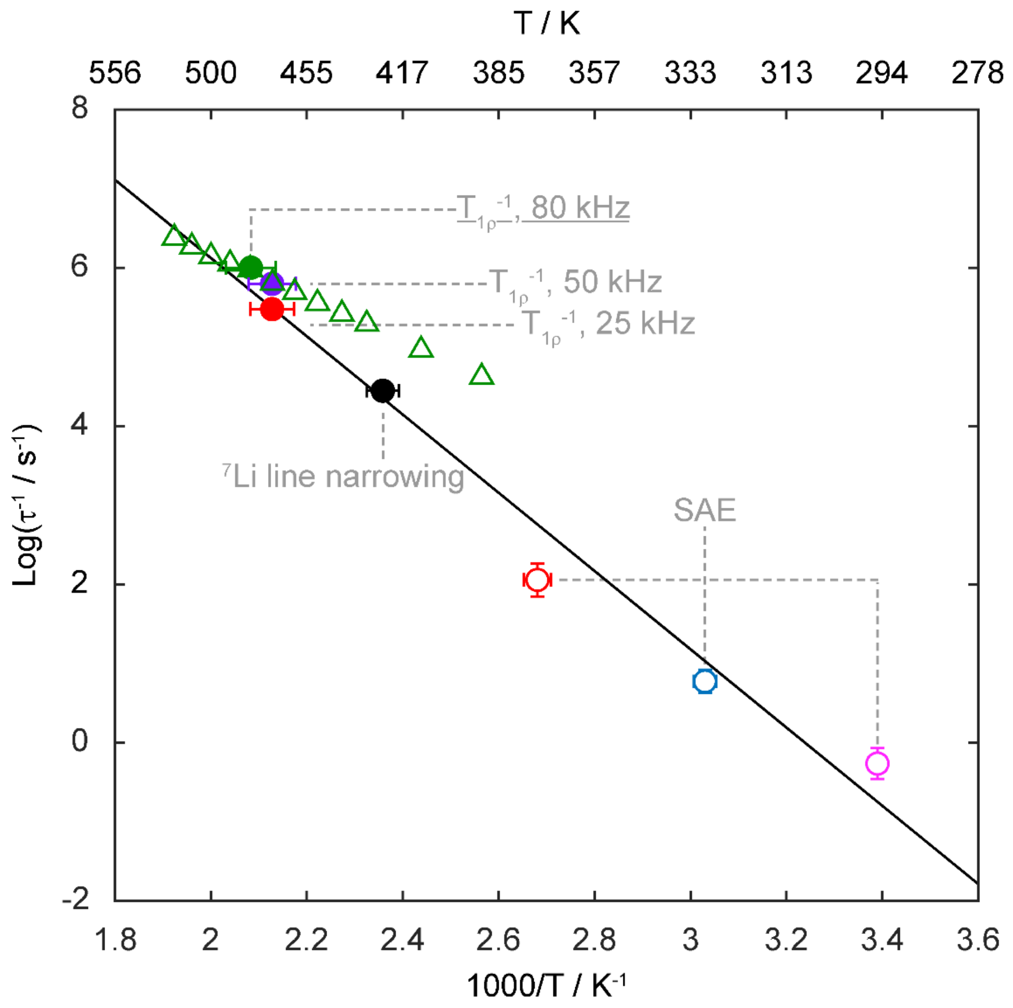

Figure S14 Arrhenius plot of Li jump rates τ_c^{-1} showing the points obtained from BPP simulations (green triangles, $\omega_1/2\pi = 80$ kHz) Data were extracted from the onset of ^{7}Li line narrowing of the variable temperature ^{7}Li NMR spectra (black circle, previously reported data), ^{7}Li SAE experiments (empty coloured circles, **Figure 9**), SLR rates in the rotating frame ($T_{1\rho}^{-1}$) experiments (filled coloured circles, **Figure 7**) at spin lock frequencies $\omega_1/2\pi$ of 25 (red), 50 (purple) and 80 kHz (green), respectively. The label for the spin locking frequency used in the BPP simulation for this figure is underlined. Errors in the jump rate τ_c^{-1} are within the data points.

References

(1) Ivanov-Shitz, A. K.; Kireev, V. V.; Mel'Nikov, O. K.; Demianets, L. N. Growth and ionic conductivity of γ-Li_3PO_4. *Crystallography Reports* **2001**, *46*, 864.
(2) Oudahmane, A.; Mbarek, A.; El-Ghozzi, M.; Avignant, D. Aluminium cyclohexaphosphate. *Acta Crystallographica Section E Structure Reports Online* **2010**, *66*, i17.
(3) Guitel, J. C.; Tordjman, I. Structure cristalline de polyphosphate de lithium $LiPO_3$. *Acta Crystallographica Section B Structural Crystallography and Crystal Chemistry* **1976**, *32*, 2960.
(4) Han, G.; Vasylenko, A.; Neale, A. R.; Duff, B. B.; Chen, R.; Dyer, M. S.; Dang, Y.; Daniels, L. M.; Zanella, M.; Robertson, C. M.; Kershaw Cook, L. J.; Hansen, A. L.; Knapp, M.; Hardwick, L. J.; Blanc, F.; Claridge, J. B.; Rosseinsky, M. J. Extended Condensed Ultraphosphate Frameworks with Monovalent Ions Combine Lithium Mobility with High Computed Electrochemical Stability. *Journal of the American Chemical Society* **2021**, *143*, 18216.
